# Baseline Goblet Cell Mucin Secretion in the Airways Exceeds Stimulated Secretion over Extended Time Periods, and Is Sensitive to Shear Stress and Intracellular Mucin Stores

**DOI:** 10.1371/journal.pone.0127267

**Published:** 2015-05-29

**Authors:** Yunxiang Zhu, Lubna H. Abdullah, Sean P. Doyle, Kristine Nguyen, Carla M. P. Ribeiro, Paula A. Vasquez, M. Gregory Forest, Michael I. Lethem, Burton F. Dickey, C. William Davis

**Affiliations:** 1 Cystic Fibrosis/Pulmonary Research and Treatment Center, University of North Carolina, Chapel Hill, North Carolina, 27599–7248, United States of America; 2 Department of Cell Biology and Physiology, University of North Carolina, Chapel Hill, North Carolina, 27599–7545, United States of America; 3 Department of Mathematics, University of North Carolina, Chapel Hill, North Carolina, 27599–3250, United States of America; 4 Pharmacy and Biomolecular Sciences, University of Brighton, Brighton, Moulsecoomb, BN2 4GJ, United Kingdom; 5 Department of Pulmonary Medicine, University of Texas M. D. Anderson Cancer Center, Houston, Texas, 77030, United States of America; University of Alabama at Birmingham, UNITED STATES

## Abstract

Airway mucin secretion studies have focused on goblet cell responses to exogenous agonists almost to the exclusion of baseline mucin secretion (BLMS). In human bronchial epithelial cell cultures (HBECCs), maximal agonist-stimulated secretion exceeds baseline by ~3-fold as measured over hour-long periods, but mucin stores are discharged completely and require 24 h for full restoration. Hence, over 24 h, total baseline exceeds agonist-induced secretion by several-fold. Studies with HBECCs and mouse tracheas showed that BLMS is highly sensitive to mechanical stresses. Harvesting three consecutive 1 h baseline luminal incubations with HBECCs yielded equal rates of BLMS; however, lengthening the middle period to 72 h decreased the respective rate significantly, suggesting a stimulation of BLMS by the gentle washes of HBECC luminal surfaces. BLMS declined exponentially after washing HBECCs (*t_1/2_* = 2.75 h), to rates approaching zero. HBECCs exposed to low perfusion rates exhibited spike-like increases in BLMS when flow was jumped 5-fold: BLMS increased >4 fold, then decreased within 5 min to a stable plateau at 1.5–2-fold over control. Higher flow jumps induced proportionally higher BLMS increases. Inducing mucous hyperplasia in HBECCs increased mucin production, BLMS and agonist-induced secretion. Mouse tracheal BLMS was ~6-fold higher during perfusion, than when flow was stopped. Munc13-2 null mouse tracheas, with their defect of accumulated cellular mucins, exhibited similar BLMS as WT, contrary to predictions of lower values. Graded mucous metaplasia induced in WT and Munc13-2 null tracheas with IL-13, caused proportional increases in BLMS, suggesting that naïve Munc13-2 mouse BLMS is elevated by increased mucin stores. We conclude that BLMS is, [i] a major component of mucin secretion in the lung, [ii] sustained by the mechanical activity of a dynamic lung, [iii] proportional to levels of mucin stores, and [iv] regulated differentially from agonist-induced mucin secretion.

## Introduction

Mucus in the airways represents the first line of innate defense in the airways against inhaled aerosols and pathogens [1]. In healthy lungs it is formed on the airway mucosa from the secretion and hydration of mucins from surface goblet cells (MUC5AC and MUC5B) and from submucosal glands (MUC5B alone). In all the inflammatory lung diseases (chronic bronchitis, asthma, cystic fibrosis, etc.), however, mucous metaplasia, hyperplasia, and hypertrophy drive mucin hypersecretion which often results in mucous plugging of the airways and other pathological conditions [2]. Because of this clinical duality, mucus and the secretion of mucins have been major areas of interest in lung biology over the last 50 or more years, increasingly so in the past decade.

In contrast to submucosal glands, where secretion appears to be regulated primarily by sympathetic and parasympathetic innervation [3,4], airway goblet cells are regulated locally by paracrine and autocrine mediators, especially ATP [5,6]. Notably, the focus of research on goblet cell mucin secretion has been on agonist-induced mucin secretion, to the virtual exclusion of consideration of mucin secretion at baseline. Retrospectively, this focus may have been short-sighted: in 11 studies from 6 different laboratories working with goblet cells in native airways or primary airway epithelial cell cultures from human and other mammalian sources [7–16], the average increase of ATP-induced mucin release was just 3.2 ± 0.5 fold higher than baseline when determined over equal periods of time (mean ± SE). This modest stimulation suggests a hypothesis that the mucins secreted at baseline may be significant, a prospect investigated in this paper.

The terms, *baseline*, *basal*, and *constitutive* secretion can all be used to indicate the release of material under control conditions, but they are also used in different contexts by physiologists and cell biologists. Since *constitutive* secretion and *basal (= ‘constitutive-like’)* secretion relate directly to different limbs of the secretory pathway [5,17] we use the term, *baseline secretion*, to describe the release of mucins *in the absence of exogenously applied agonist*. It is used without regard to a specific limb of the secretory pathway, or mechanism; however, the release of mucins is presumed to occur exclusively via the regulated secretory pathway, and not via the constitutive pathway, as evidenced by its dependency on extracellular signals, cytoplasmic Ca^2+^, and the second messenger sensing, SNARE-priming protein Munc13-2.

In mouse, the deletion of Munc13-2 resulted in a loss-of-function phenotype in the airways that was interpreted as a defect in the secretory pathway [7]. The phenotype signified the potential importance of baseline mucin secretion. The secretory cells of mouse airways, club cells (formerly Clara cells), do not stain with alcian blue/periodic acid-Schiff (AB/PAS) under control conditions, due to a paucity of stored mucins. Club cells assume a goblet cell-like phenotype in inflammation [18], and are converted to a goblet cell-like model for experimental purposes by upregulating mucin production with the induction of allergic mucous metaplasia [19,20]. In the Munc13-2 null mouse under control conditions, however, the club cells are positive for AB/PAS (AB/PAS+) which was shown to be due to an accumulation of mucin secretory granules in the absence of increased mucin production. Deletion of the Munc13-2 gene interfered with baseline mucin release, causing an accumulation in mucin stores [7]. Previously, club cells were thought to be devoid of mucin under control conditions, but this study showed that mucins are in fact produced in WT mice. Rather than being stored in significant quantities, though, the mucins are secreted at baseline.

In this paper, human bronchial epithelial cell cultures (HBECCs; see Abbreviations and definitions, S1 File) and mouse tracheas were used to test the significance of goblet cell baseline mucin secretion in the airways and to identify potential physical and humoral factors that might modulate or regulate baseline release. The data derived suggest that baseline mucin secretion is the predominant mode of goblet cell mucin release in the healthy lung, and is likely to also contribute significantly, if not predominately, to mucus and/or sputum formation in allergic and infectious inflammation.

## Methods

### Materials

ATPγS was purchased from Roche Applied Science (Indianapolis, IN); unless otherwise noted, all other chemicals were purchased from Sigma-Aldrich Chemical Co. (St. Louis, MO).

### Mouse care and experimental procedures

Munc13-2 (C57BL/6 background) and P2Y2R (129S6 background) deficient mice were received originally from Drs. Niels Brose (Max Planck Institute of Experimental Medicine, Germany; [21] and Beverly Koller [University of North Carolina, Chapel Hill, NC; [22]). The respective 129S6 WT mice were purchased from Taconic (Hudson, NY), or The Jackson Laboratory (Bar Harbor, ME), respectively. All mice were bred and raised at the University of North Carolina and allowed food and water *ad libitum*. Some animals were treated with ovalbumin (OVA) or IL-13 instillation under isoflurane anesthesia as described below; all animals were ultimately euthanized with gaseous CO_2_ prior to tissue harvest by dissection. All experimental procedures using mice were conducted under protocols approved by the University of North Carolina Institutional Animal Use and Care Committee.

### Airway mucous metaplasia

Airway mucous metaplasia was induced in mice using the OVA sensitization and challenge procedure detailed previously [11]. Briefly, OVA was injected i.p. on days 0, 7 and 14, and on days 21 and 24, 50 μl of 2.0% OVA in PBS was instilled by aspiration into tracheas of isoflurane-anaesthetized mice. Experimental procedures were performed 3–5 days following the 2nd OVA instillation. Alternately, using the same isoflurane anesthesia, mucous metaplasia was induced instead by the direct instillation of IL-13 into the trachea (recombinant, carrier free murine IL-13, (Biolegend, San Diego, CA). To achieve a variable degree of IL-13-induced metaplasia, the number of instillations and the time between the last instillation and tissue harvest were varied, as described in Results and in Table 1.

**Table 1:** IL-13 Treatments Used to Induce Graded Mucous Metaplasia.

*IL-13 Treatment*		*Day of Experiment*	*Degree of Metaplasia*	*Mucus Plugs*
# 0(control)	Day 0; 0 μg IL-13	Day 3	-	-
# 1	Day 0; 1 μg	Day 3	+	-
# 2	Day 0, 1; 2 μg each	Day 2	++	-
# 3	Day 0, 1, 2; 1 μg each	Day 3	+++	+
# 4	Day 0, 1, 2; 1 μg each	Day 5	++++	+++

Responses:— = no effect, + = visible increase, ++ = substantial increase, +++ = major increase, ++++ = extraordinary increase.

### Mouse tracheal perfusion and mucin ELISA

Tracheas harvested following CO_2_ euthanasia were canulated at their proximal ends and perfused at ~30 μl/min with warmed, 5% CO_2_/95% O_2_-equilibrated DMEM/F12 as detailed previously [11]. The entire set-up (pump, solutions, tissues, fraction collector) was housed in a humidified Nuaire DH Autoflow CO_2_ incubator (Plymouth, MN, USA), factory customized to lie horizontally. Mucins were collected into the wells of 96-well, non-binding, polystyrene microtiter storage plates (# 3594, Corning, Tewksbury, MA). Following an experiment, the plates were tightly sealed and frozen until assessed for mucins in the collected fractions using an ELISA.

The ELISA followed the procedures developed in this laboratory and described in a recent methods paper [23]. It used a mucin subunit antibody, a polyclonal antibody that recognizes all vertebrate polymeric mucins [11,23,24] (the antibody was a gift of Dr. David Thornton). Standard curves were generated from mucins purified from sputum collected from patients with cystic fibrosis [23] applied to each plate and analyzed by fitting with the Four Parameter Logistic Equation using a data acquisition and analysis software package, *SoftMax Pro*, that accompanied the SectraMax Plus plate reader (Molecular Devices, Sunnyvale, CA). The results were expressed as the equivalent nanogram of mucin released per each fraction for each trachea. Data generated from this assay are identified in figure legends as having been derived with the ‘subunit ELISA’. Assay sensitivity for these tracheal perfusions was increased by minimizing assay-to-assay variability, by assessing all the samples for an experiment at the same time. Potential statistical differences between sample means in these experiments were subjected to a Student’s t test, assuming equal variances, with statistical significance being indicated at *p* <0.05.

### Histology and microscopy

Human and mouse tissues were fixed in formalin, dehydrated and embedded in paraffin, and sections cut at 5 μm were placed on slides, deparaffinized, rehydrated, and stained with AB/PAS, using a 5 min incubation in 0.5% periodic acid, following standard protocols. Where necessary, mucous metaplasia in mouse lungs was quantified from images of the left interlobar bronchus taken with an upright Nikon Microphot-SA microscope interfaced with a DXM 1200 color camera (Nikon Instruments) at 10X magnification. The AB/PAS-positive area was determined using ImageJ image processing software (http://rsb.info.nih.gov/ij/) to threshold grayscale images, expressing the integrated density of the area of AB/PAS+ mucosubstances per unit length of basement membrane [25,26].

### Human bronchial epithelial cell culture, mucin biochemistry, and mucin secretion

HBE cells were obtained in accordance with Institutional Review Board-approved protocols, as described previously [23,27], from normal human bronchi. Briefly, HBE cells were isolated and grown on plastic culture dishes in bronchial epithelial cell growth medium and passaged at 80% confluence, and first-passage cells were seeded onto 12-mm Transwell-Clear supports (TClears; Corning) at 250,000 per support. At confluence, the cells were maintained under air-liquid interface (ALI) conditions in ALI culture medium (bronchial epithelial cell growth medium modified per Ref [27]), which was changed at the basolateral surface three times a week. HBECCs were used for experiments 4–6 weeks post-confluence, a time when the columnar cells are well differentiated as ciliated and goblet cells.

MUC5B in HBECCs was analyzed by Western blotting following extraction with 300 μl of the extraction buffer in the RNeasy Mini Kit (Cat. #74104, Qiagen). Since this is a guanidine-based buffer, it serves equally well for the extraction of nucleic acids and mucins from HBECCs [28,29]. The sample extracts were prepared for electrophoresis on 1% agarose gels as previously described [30,31]. After electrophoretic separation and vacuum blotting of the mucins onto nitrocellulose, the mature and immature forms of MUC5B in the immunoblots were probed semi-quantitatively with the following specific antibodies:

*MUC5B—immature peptide*, 5BVNTR-1, a rabbit polyclonal antibody generated for this study that recognizes a sequence (SSPGTATALPALRSTATTPTATS) in the PTS/mucin tandem repeat domains of MUC5B prior to glycosylation (restricting detection to MUC5B peptides formed in the ER, before transit to the Golgi complex); and,

*MUC5B—mature glycoprotein*, EU-MUC5B, a mouse IgG1, (a gift from Dr. David Thornton, University of Manchester), that recognizes the peptide sequence, RNREQVGKFKMC, located in the globular, Cys-rich subdomains within the PTS/mucin tandem repeat domains [32].

**Fig 1:**
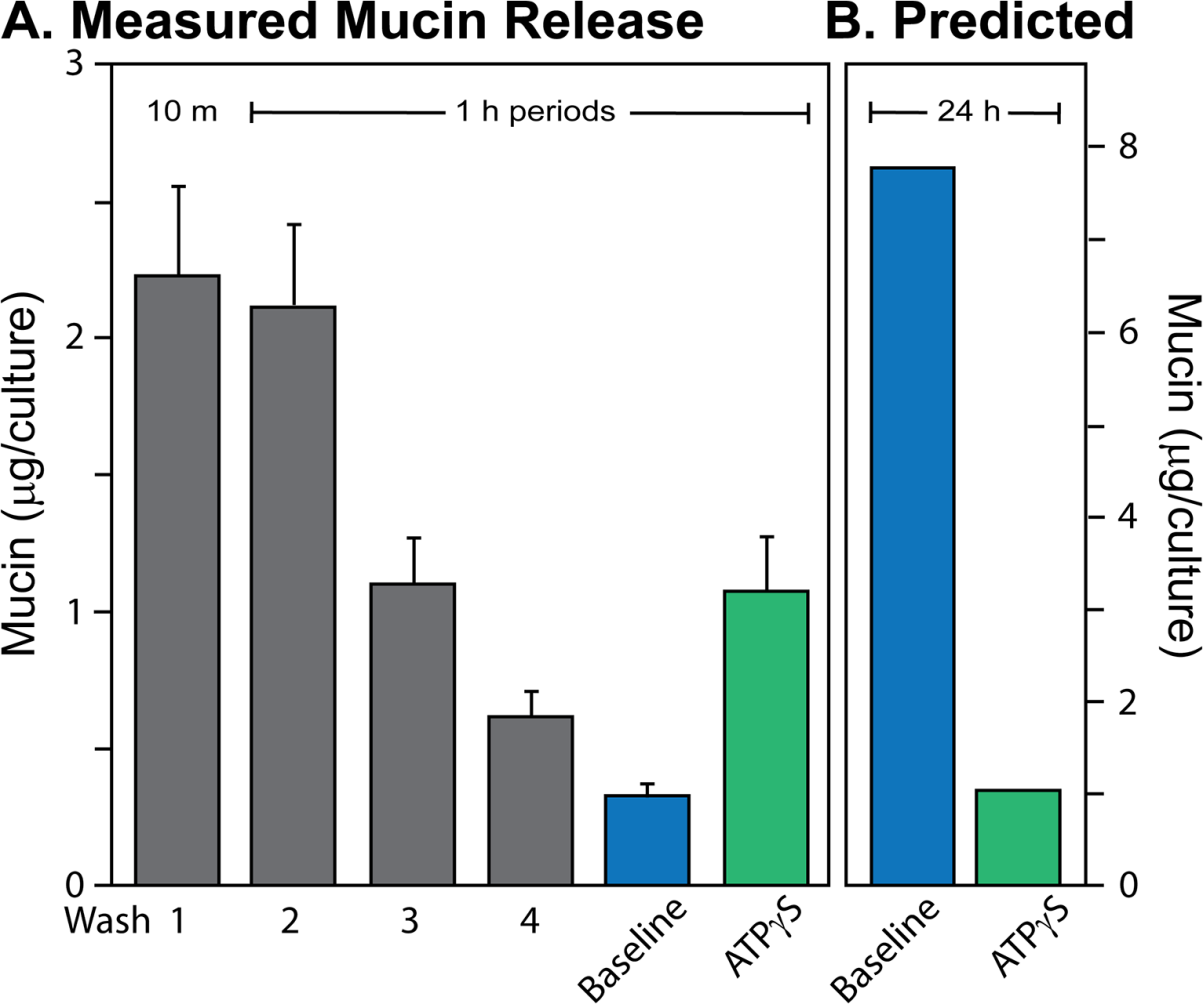
Mucin secretion from HBECCs. ***A*. *Measured mucins*.** Mucins were assessed by subunit ELISA for the accumulated mucus removed from HBECCs with a series of Careful Washes (Wash 1, 10 min, Wash 3–4, 1 h), and from the secretions released during 1 h baseline and agonist stimulation (100 μM ATPγS) periods. Data are expressed as the mean ± SE, n = 10 sets of HBECCs. ***B*. *Predicted mucin release*.** Mucin release predicted for a 24 h day from the data in Panel A. Predicted baseline was estimated by extended the 1 h baseline out to 24 h. Agonist-induced secretion over 24 h was estimated as just that released acutely (see text). Note the different scales, left and right.

Mucin secretion experiments with HBECCs under static, i.e., non-perfused, conditions were conducted as detailed previously [23]. Briefly, the mucus which accumulates on the luminal surfaces HBECCs in the 2–3 days between culture maintenance under ALI conditions was removed with a ‘Careful Wash’, a series of 4 luminal washes rigorously controlled to minimize mechanical vibration and shear forces associated with replacement of luminal liquid. Each ‘wash’ consisted of the addition of 0.5 ml DMEM/F12 culture medium warmed to 37°C and either a 10 min (1^st^ wash) or 60 min (washes 2–4) incubation. The liquids removed were saved and the contained mucins determined along with the mucins later secreted during the experiments (see Fig 1 and [23]). The cultures were then used in the experiments, as detailed in Results. Mucins in the luminal liquids removed from the cultures were assessed by ELISA using the same mucin subunit’ antibody used for mouse tracheal samples (above), and, as above, data generated from this assay are identified in figure legends as having been derived with the ‘subunit ELISA’.

The high resolution video microscopy and mucin secretion data of Fig 6A were collected in 1993, following previously detailed procedures [33].

### Perfusion of HBECCs and mucin ELLA

HBE cells were grown as above, on 24-mm TClears, beginning with a plating density of 1 million per support. At the time of the experiments, the cultures were removed from the incubator and the luminal surface was washed with a single volume of DMEM/F12 medium before being fitted with a gasket made from 2 mm Neopreme material. The gasket had a 10 x 16 mm rounded-rectangular cutout that defined a perfusion slot (volume = 270 μl; see S1 Fig). Next, a perfusion plug with an O-ring seal was inserted into the TClear (see [9]) which allowed perfusion of the HBECC over a 2 mm high area defined by the gasket. The cultures were perfused at 100 μl/min with DMEM/F12 medium using a Harvard Apparatus (Holliston, MA) PHD 2000 syringe pump during a 2 h equilibration period, following which 1 min fractions were collected during a 10 min baseline secretion period. The perfusion rate was then elevated in a single step to a higher rate, as described in Results, with fractions continuing to be collected at 1 min intervals. After the experiment, the mucins contained in the fractions were determined with an ELLA, using wheat-germ agglutinin (WGA) to detect the mucins, as described previously [23]. Data generated from this assay are identified in figure legends as having been derived with the ‘WGA ELLA’. The shear stresses (τ_ω_) due to flow at the epithelial surface, expressed as millidynes/cm^2^ (mdyn/cm^2^) were estimated from flow through a rectangular slot, consistent with Poiseuille flow (for which the slip velocity at the cell surface was assumed to approximate to 0):

$$\tau_{\omega }=6\eta Q/wh^{2}\;,$$

where

η = viscosity, and is assumed equal to water, 0.001 Pa-s;

Q = volume flow rate, cm^3^/s; and,

w = width of the channel, 0.85 cm;

h = height of the channel, 0.19 cm.

Similarly, shear stresses imposed during the perfusion of mouse tracheas were calculated for Poiseuille flow through a cylinder, as,

$$\tau_{\omega }=4\eta Q/\pi r^{3},$$

where

r = radius, taken as a nominal 0.1 cm.

Poiseuille flow was assumed based on Reynolds Numbers (Re = 4Qρ/ ηP, where ρ = density and P = channel perimeter) for the flows used in our experiments (100 to 1,000 μl/min): Re ranged from 0.48 to 4.80, well below the threshold of 2040 for turbulent flow.

## Results

### Baseline secretion from HBECCs

#### Baseline versus agonist-stimulated mucin secretion in HBECCs

As background, these first studies began with a methodological effort to define a rigorous, but practical, wash procedure for HBECCs that would minimize mechanical stress during removal of accumulated mucus, while preserving goblet cell mucin stores [15,23]. This procedure now allows a comparison of the relative amounts of mucin secreted at baseline and during agonist stimulation of HBECCs (ATPγS, 100 μM) [5], as well as the mucins removed from the luminal surfaces during preparatory wash periods (i.e., a ‘Careful Wash’). Fig 1A shows that the mucins released by agonist, 1.1 ± 0.2 μg/culture, were 3.3-fold higher than baseline release, both measured over 1 h periods, consistent with previous determinations [10,12,14–16]. Not only was the ratio of baseline/stimulated mucin release modest, but the quantity of mucins removed from the cultures prior to the experiment, 5.98 μg, exceeded those released in response to agonist by 5.7-fold. These data are consistent with a relatively robust release of mucins at baseline; however, any conclusion regarding the relative rates of baseline and agonist-induced mucin secretion over macroscopic periods of time requires knowing the time required for intracellular mucin store recharge following stimulation by agonist.

**Fig 2:**
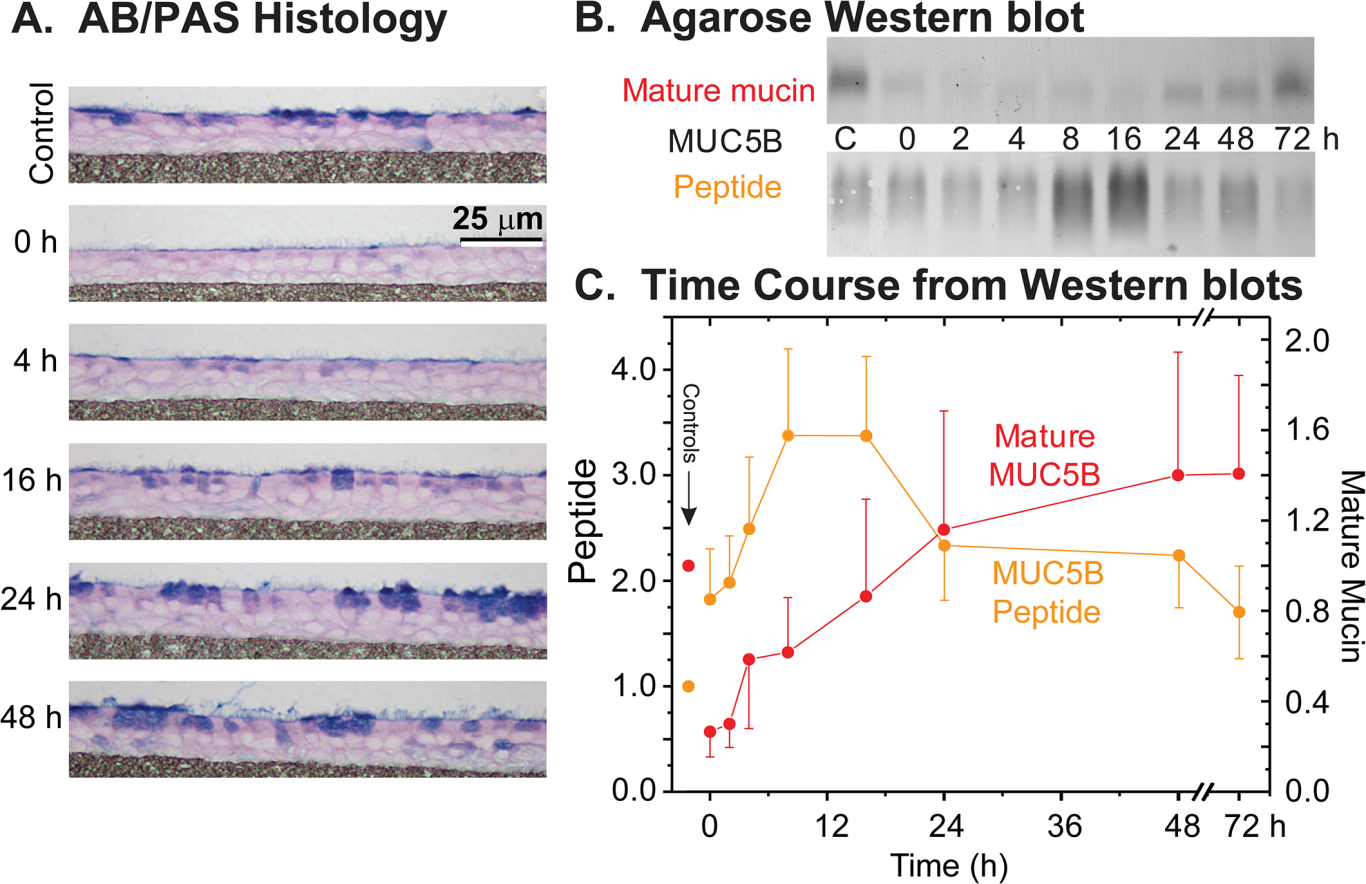
Recovery of mucin stores in HBE cell cultures following agonist (ATPγS, 100 μM) induced discharge. ***A*. *HBECCs examined by AB/PAS histology*.** HBECCs were fixed before (control) and periodically after exposure to agonist, and mucin stores were revealed by AB/PAS staining. ***B*. *Sample agarose Western blots*.** Whole cell extracts of HBECCs sampled at the indicated times were subjected to electrophoresis in agarose gels, vacuum blotted, and the blots probed for MUC5B with domain-specific antibodies. ‘Immature’, non-glycosylated mucin peptides were detected with an antibody to the PTS/mucin repeat domains, and ‘mature’, fully glycosylated mucins with antibodies to the Cys-rich domains within the glycosylated mucin domains. ***C*. *Time course of MUC5B peptides and mature mucins*.** Mucin quantitation was achieved by densitometry of agarose Western blots as in Panel B; all results are expressed relative to their respective controls. Left axis = MUC5B peptide as the immature, non-glycosylated form of the mucin, right axis = mature MUC5B glycoprotein. Time 0 = 45 min post-ATPγS, after cultures were washed to remove the secreted mucus; Controls (‘C’ in Panel B) = non-treated HBE cells. Data expressed as the mean ± SE (n = 7–9 sets).

To determine the recovery time for mucin stores, HBECCs were washed free of accumulated mucins, stimulated for 1 h with 100 μM ATPγS, and cultures were then periodically fixed for histology or extracted for total mucin determinations for up to 72 h post-discharge. Consistent with the original observations of Kemp, et al [15], maximal stimulation with P2Y_2_ agonist caused a full discharge of HBECC mucin stores, relative to control, as is apparent at t = 0 by the paucity of AB/PAS staining and by the minimal staining of agarose Western blots stained for the mature, glycosylated form of MUC5B (Fig 2A and 2B). The recovery of mucin stores was followed semi-quantitatively by Western blotting (Fig 2B and 2C), staining for mature MUC5B, as well as for the non-glycosylated MUC5B peptide. The latter is taken as an index of mucin synthesis since this form of MUC5B occurs only in the ER [34]. From these data, it is clear that several hours are required following a full mucin discharge for a significant recovery of mucin stores: mucin synthesis appeared to peak as a broad plateau, 8–16 h post-discharge, and the half-time of mucin store recovery was 8–12 h post-discharge (Fig 2C). Full recovery of mucin stores required ~24 h.

With the time required for mucin store recharge established, the data in Fig 1A were used to estimate the relative amounts of mucin secreted over the period of a day by baseline and agonist-induced secretory modalities (Fig 1B). The mucins secreted over 24 h during agonist stimulation were taken as those released during a 1 h exposure to ATPγS, since the treatment fully exhausts mucins stores and a complete recovery takes the better part of a day (Fig 2). The mucins secreted at baseline were estimated by projecting the 1 h rate of release out over 24 h, since the release presumably reflects a steady-state activity of the goblet cells. When viewed this way (Fig 1B), baseline mucin secretion is predicted to exceed agonist induced mucin secretion by >7-fold over the course of a day. This, of course, is an overestimate because agonist-stimulated goblet cells achieve a full recharge in less than 24 h, and are presumably capable of further agonist-induced secretions. However, if one doubled the mucins secreted in response to agonist, the amount would still be exceeded by mucins secreted at baseline each day by ~3.5 fold. The calculation therefore suggests that the baseline release of mucins over the period of a day is potentially more substantial than a full, acute mucin release in response to agonist. Baseline mucin release may therefore be an important, or predominant mucin secretory modality for the healthy lung.

#### Sensitivity of baseline HBECC mucin secretion to experimental handling

**Fig 3:**
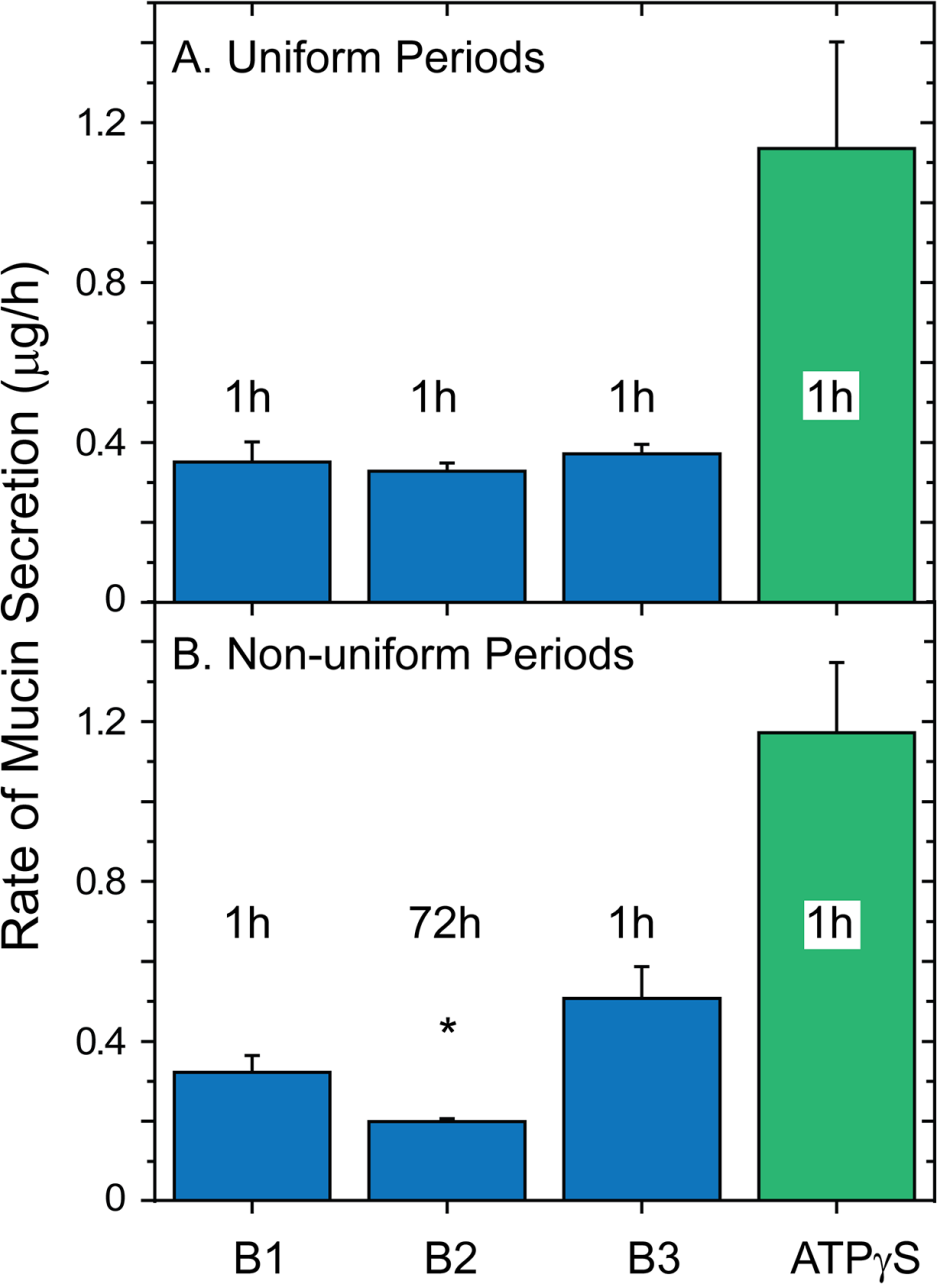
Measured baseline secretion rates vary with period duration. Following the Careful Wash protocol of Fig 1, HBECC mucin secretion rates were determined for three successive baseline periods, then the cultures were exposed to agonist (100 μM ATPγS). ***A*. *Uniform baseline periods***. The baseline periods were all 1 hr in duration. ***B*. *Non-uniform baseline periods*.** The first and last baseline periods were 1 hr, but the middle one was 72 hr in duration. After the cultures were sampled for the B1 period, they were returned to the incubator for the 72 hr B2 period, and the mucins released were harvested by performing a 2^nd^ Careful Wash and combining the samples. n = 6; * *p < 0*.*05* relative to period B1. Mucins in all samples were determined by subunit ELISA.

Airway epithelia are very sensitive to mechanical stresses, including the shear stresses associated with luminal media changes and perfusion (e.g., see [35,36]). Consequently, we assessed the effects of handling on HBECCs during experiments designed to measure baseline secretion, initially performing two experiments similar to that in Fig 1. First, following a Careful Wash, the series of washes preceding a baseline incubation period (see Fig 1), mucin release was determined during 3 successive periods of equal, 1 h incubations, and again in the presence of agonist (ATPγS, 100 μM). The result (Fig 3A) showed that mucin secretion in all three periods of baseline secretion were similar to one another, and the agonist-stimulated release was ~3-fold higher, as it is when preceded with a single baseline period (c.f., Figs 1A and 3A).

Second, the protocol above was followed with the exception that the second baseline period was 72 h in duration (Fig 3B). Because HBECCs cannot tolerate 0.5 ml of luminal medium for more than 6–8 h during experiments such as these (the liquid becomes acidic and the epithelium loses its integrity), luminal liquid was not added following harvest of the liquid for the first baseline period. Instead the cultures were returned to the air:liquid conditions of the incubator. After 71 h, 0.5 ml of media was added to the cultures and harvested 1 h later for the second baseline period, with the experiment then being completed following normal procedures. In this case, the rate of baseline mucin secretion (μg/h) during the second period was depressed significantly relative to control (period B1, Fig 3B). In absolute terms, more mucins were released in the middle, 72 h period, but the larger amount was released over a much longer period of time. Mucin release during the third baseline period rebounded and had a rate similar to the control period, and agonist elicited its usual ~3-fold stimulation (Fig 3B).

**Fig 4:**
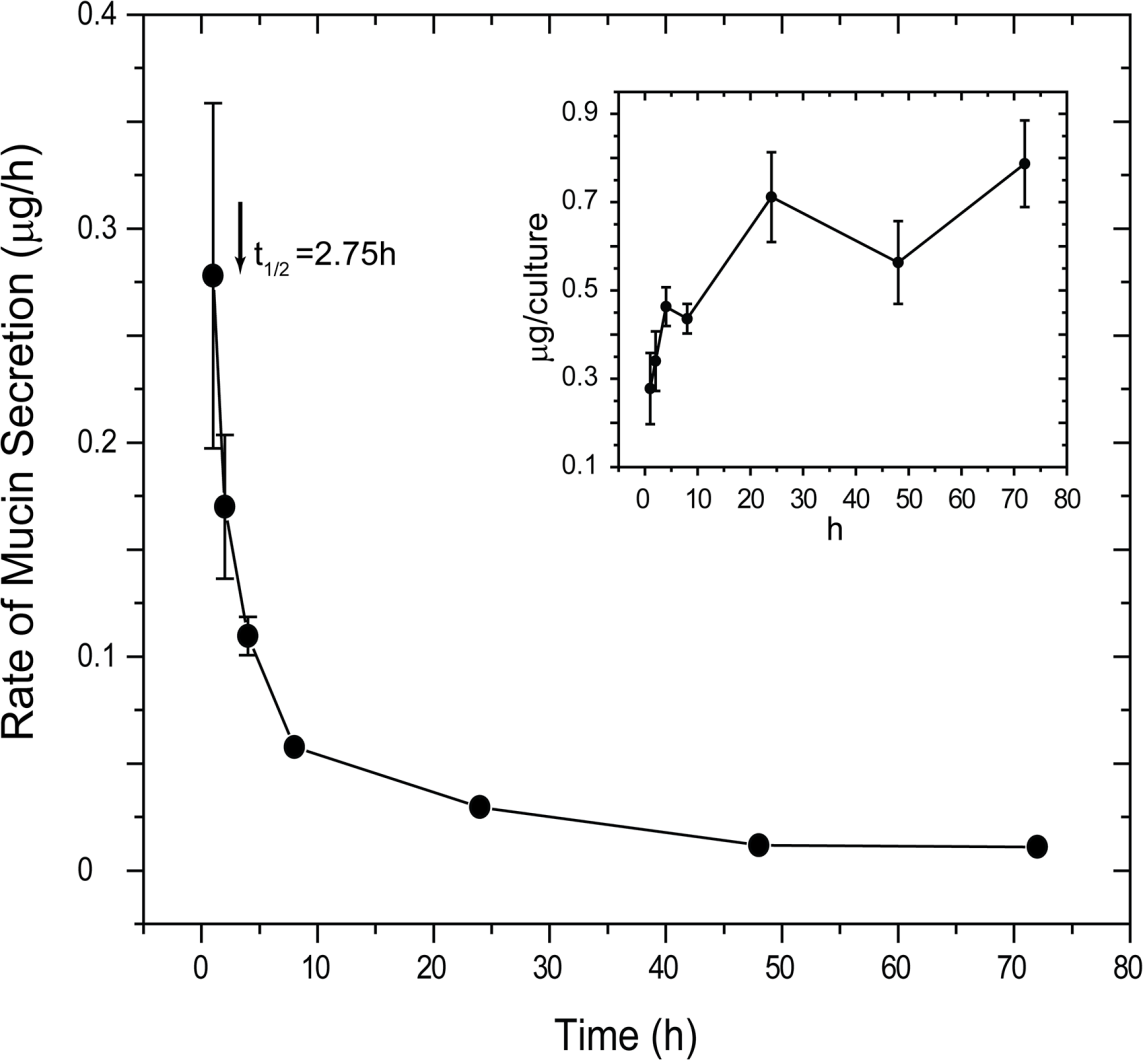
Time course of changes in HBECC baseline mucin secretion following a Careful Wash. HBECCs were Carefully Washed and returned to the incubator under air:liquid luminal conditions. The cultures were then removed as a function of time and luminal mucus harvested with minimal perturbation. Cultures were sampled just once; different sets of cultures were used at the different time points (n = 4). *Inset*: Data plotted to show the total mucins harvested from the cultures over time. Mucins in all samples were determined by subunit ELISA.

The results above suggested that HBECCs are sensitive to the handling necessary to add and remove luminal media, including the mechanical stresses associated with transferring the cultures between incubator and tissue culture hood. To test this sensitivity more rigorously, the time course of the potential relaxation in baseline mucin secretion was determined under conditions in which handling of the cultures was minimized. Four sets of HBECCs, 7 cultures/set, were Carefully Washed and returned to the incubator under luminal air:liquid conditions. Beginning 1 h later, the first culture of each set was removed from the incubator, 0.5 ml of media was added to the lumen, incubated 10 min, and removed for assessment of mucins released during the intervening period. The other cultures in a set were treated similarly at 2–72 h, with the mucins collected from each culture at just a single time point. As shown in the inset to Fig 4, mucins accumulated on the luminal surfaces of the HBECCs for the first few hours following the wash, but then approached an apparent plateau. When plotted as a rate, baseline mucin secretion decreased dramatically following a wash, reaching a stable minimum after 24 h; the half-time of the relaxation was 2.75 h (Fig 4). Consequently, the appearance of a stable baseline rate of secretion for the series of uniform 1 h sample periods shown in Fig 3A most likely resulted from the periodic handling of the cultures necessary to harvest the mucins at the end of each period. Also, washing a HBECC in a standardized manner appears to induce a unitized response (Fig 3A and 3B).

#### Mucin secretion from perfused HBECCs

**Fig 5:**
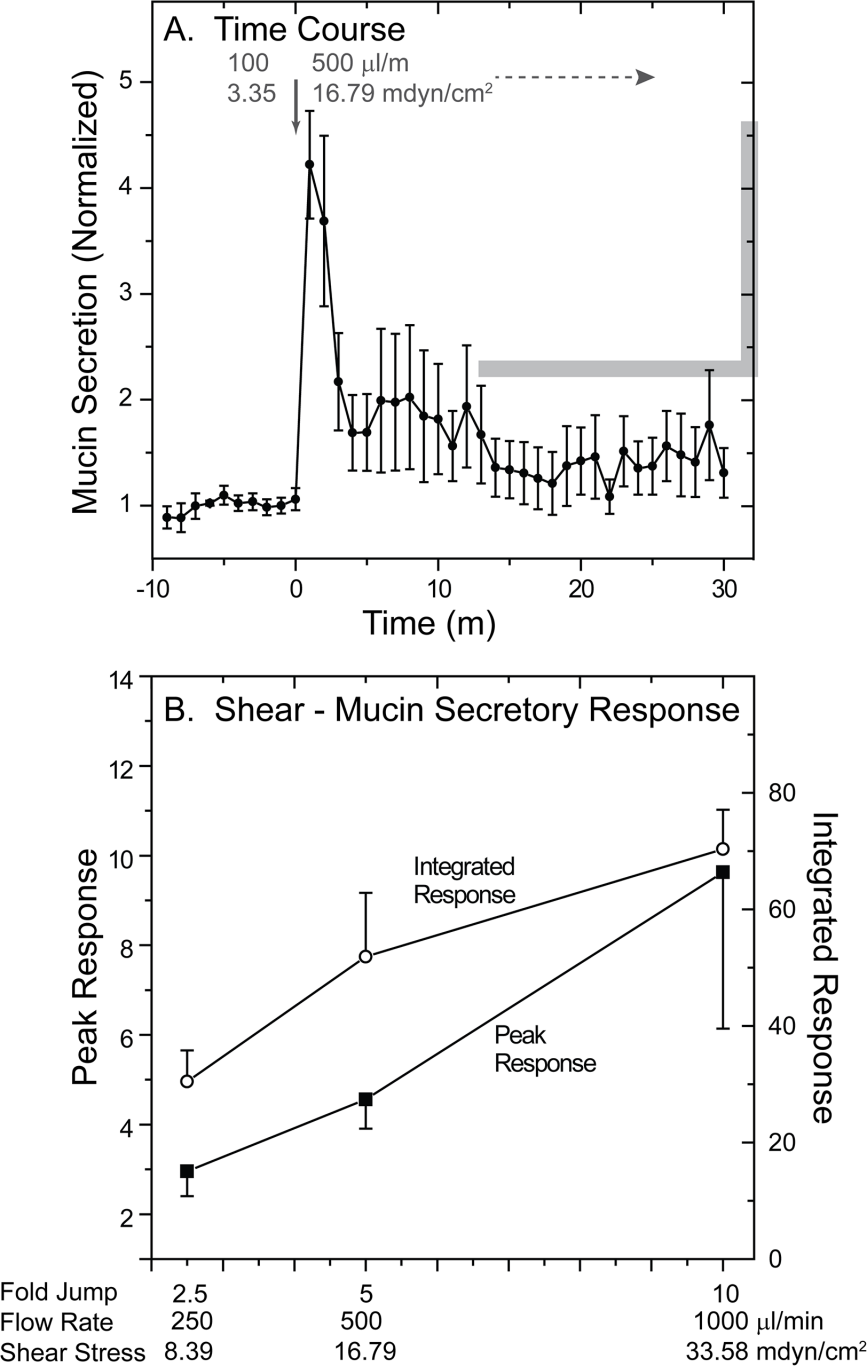
Mucin secretory response of HBECCs to liquid shear stress. HBECCs were perfused luminally at 100 μl/min during 2 h equilibration and 10 min baseline collection periods, then the perfusion rate was increased in a single step (arrow) by 2.5, 5, or 10 fold. Fractions collected during the baseline and increased flow periods were assessed for secreted mucins by the WGA ELLA. ***A*. *Time course showing the effects of a 5-fold increase (jump) in flow rate*.** Mucin secretion is plotted as mucin content/fraction, normalized to baseline (mean ± SE, n = 5). **B. *Relationship between the magnitude of the change in flow rate*, *and the normalized peak and integrated mucin secretory responses*.** The normalized peak and 30 min, integrated mucin secretory responses from the cultures subjected to different changes in flow-induced shear stress are plotted against the jump in flow rate. The abscissa shows the final flow rates, fold jump in flow, and the calculated, final steady-state shear stresses at each final flow rate used. Each point represents the mean ± SE of 5 or more experiments.

The results of the previous experiments suggested that HBECCs are very sensitive to the mechanical stresses associated with handling and pipetting. To assess the mechanical forces required to induce an elevation of mucin secretion at baseline, we determined the sensitivity of mucin secretion from perfused HBECCs to changes in perfusion flow rate. The lumens of HBECCs grown on large, 25 mm diameter TClear supports were perfused at a constant 100 μl/min flow rate for a 2 h equilibration period (S1 Fig), and 1 min fractions then collected for a 10 min baseline period. A flow rate of 100 μl/min, corresponding to a shear stress of 3.35 mdyn/cm^2^, was selected as the initial perfusion rate, as preliminary experiments indicated minimal effects of the associated with flows of this magnitude. By comparison, this level of shear stress is on the order of 100-fold less than that associated with airflow during tidal breathing, estimated at ~450 mdyn/cm^2^ [37], but it is in the middle of the range known to stimulate the release of ATP in HBECCs [38,39]. After collection of the fractions representing the baseline flow rate, the HBECC perfusion rate was then elevated, or jumped, in a single step. As an example, Fig 5A shows the mucin secretory response to a sudden increase in flow rate from 100 to 500 μl/min (3.35 to 16.79 mdyn/cm^2^). Mucin secretion increased >4-fold coincident with the increase in flow and then declined to a plateau level elevated with respect to the baseline rate of release, despite the higher flow rate.

Fig 5B depicts the results from experiments in which perfusion flow rates were increased over a range of 2.5 to 10-fold over the baseline flow of 100 μl/min, showing the peak and integrated portions of the secretory responses observed. Interestingly, there is a linear relationship for both the peak and integrated portions of the mucin secretory responses observed and the increase in flow rate. Preliminary results examining increases less than 2.5-fold elicited weak, uncertain mucin secretory responses, while those greater than 10-fold elicited high, but erratic responses. The results suggest that mucin secretion from human airway epithelial cells is very sensitive to the stresses associated with fluid flow, stresses that are well below the range estimated for the airways during tidal breathing [37], but within the range that stimulate ATP release [38,39].

#### Time course of agonist-stimulated mucin secretion from HBECCs

**Fig 6:**
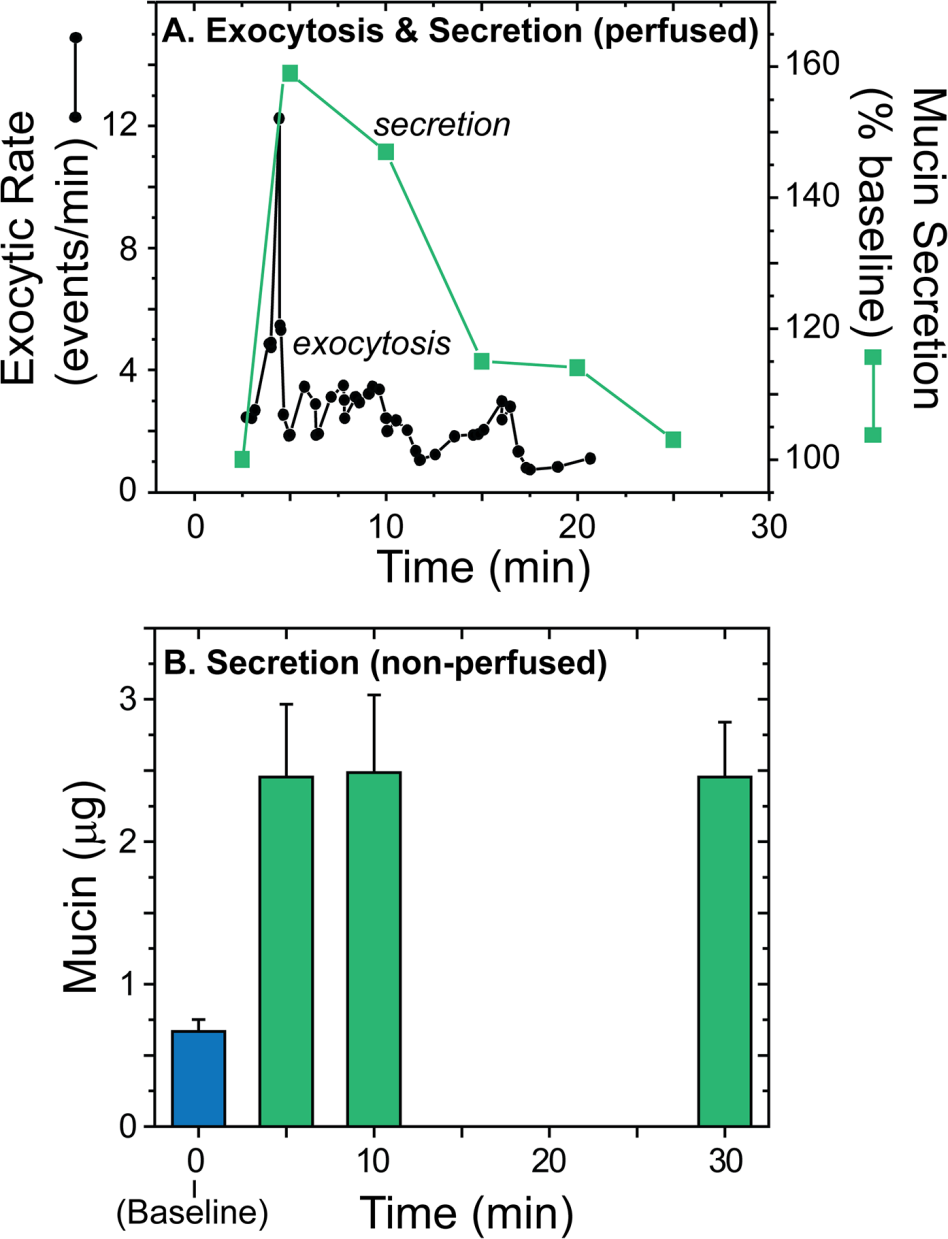
Time course of agonist-induced mucin secretion from human airways. ***A*. *Comparing exocytosis (left axis) and secretions (right axis) in an individual perfused preparation*.** Analysis of the secretory response of a single, luminally perfused human nasal epithelial explant, circa 1993, measuring exocytic events in an individual goblet cell by microscopy, and mucin secretion from the same explant by ELISA (see [33]; shear stress ~66 mdyn/cm^2^). The time course of mucin secretion was corrected for the perfusion delay. Note the short duration of the exocytic burst, as measured by microscopy, and the relatively prolonged time course of mucin secretion determined by the ELISA. ***B*. *Time course of mucin secretion from HBECCs (non-perfused)*.** Following determination of mucin secretion during a baseline period (blue bar; 30 min; n = 17), the mucins secreted in response to agonist (ATPγS, 100 μM) were determined by subunit ELISA as a function of time (green bars; n = 3–5 cultures/time point). Individual cultures were used for each time point. Note that the secretory response appears to be complete in the first 5 min.

Following stimulation of goblet cells by agonist, mucin granule exocytosis as determined directly by microscopy occurs with a burst of exocytic events (EE) that lasts < 1 min, followed by a plateau lasting a few minutes [33,40], essentially until the cell exhausts its granule stores (Fig 2A). When agonist-stimulated mucin secretion is determined in perfused preparations using biochemical assays, however, the secretory response appears to last for ~20 min or more [10,33]. Fig 6A illustrates this discrepancy with data derived, *circa* 1993, from a single nasal epithelial explant in which an individual goblet cell was observed by microscopy while mucins secreted from the entire explant were collected and analyzed by ELISA (per [33]). At the single-cell level the exocytic response was brief, while mucins were collected in significant quantities for 10–20 minutes.

To test whether the apparent discrepancy between the patterns of exocytosis and mucin release in Fig 6A was due to the secreted mucins being delayed in their exit by interactions with the walls of the perfusion chamber and tubing, a series of non-perfused HBECCs were stimulated with ATPγS and the luminal liquid was collected at 5, 10, or 30 min. As shown in Fig 6B, the quantity of mucins recovered after stimulation was independent of the time of collection, indicating that the secretory response was effectively complete in < 5 min.

#### Mucin secretion and stores in HBECCs

**Fig 7:**
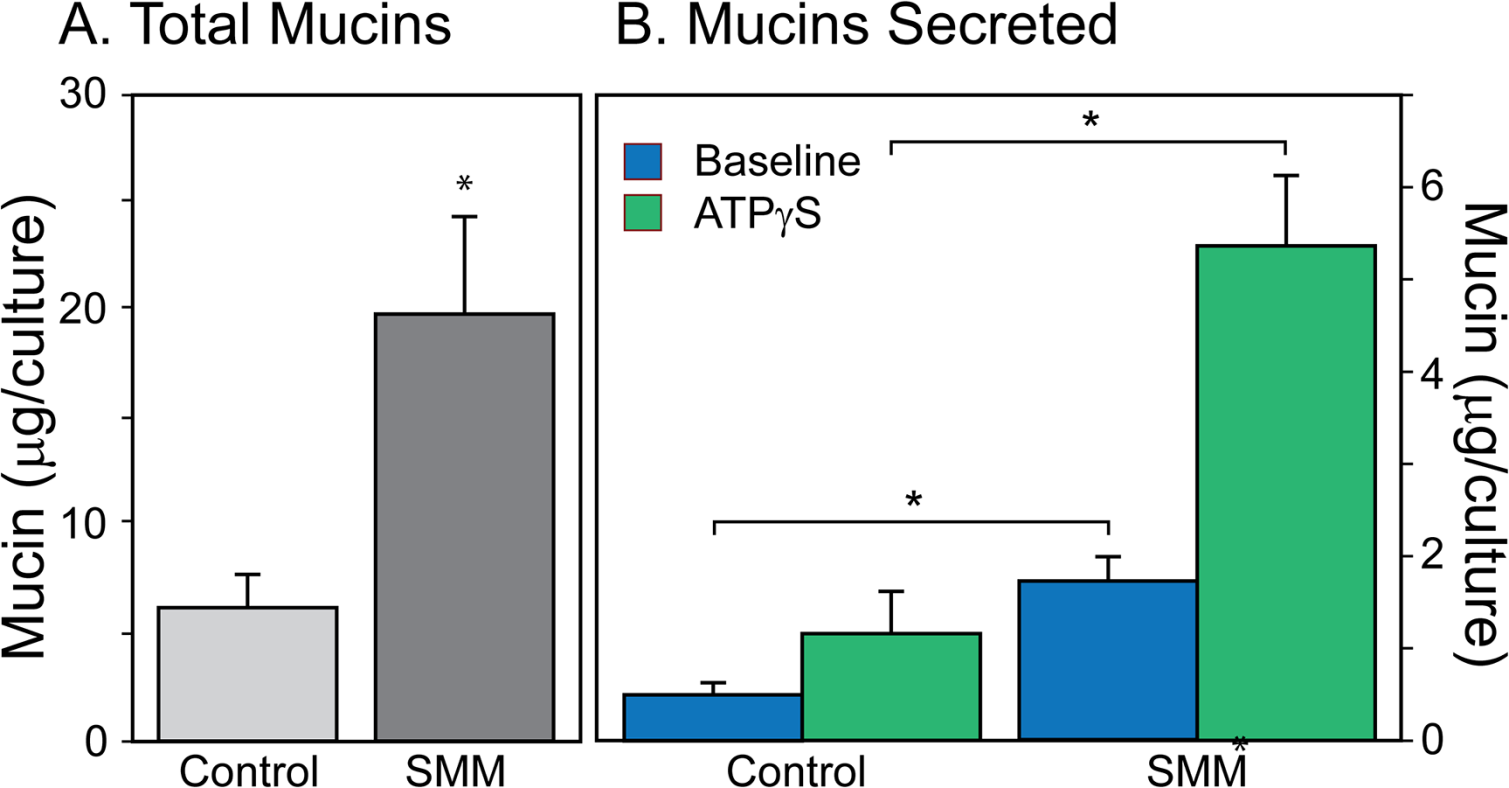
Chronic effects of SMM (72 h) on HBECCs. ***A*. *Effects on total mucins*.** Total mucins removed during the Careful Wash, and during the baseline and agonist-stimulated secretion periods, were determined individually, then summed. ***B*. *Effects on freshly secreted mucins*.** Mucins secreted at baseline and in response to ATPγS (100 μM, 45 min; * *p* < 0.05, n = 5)). Note the different ordinate scales in A and B. Mucins in all samples were determined by subunit ELISA.

If baseline mucin secretion is an important secretory modality under control conditions, as the results above suggest, it is also important to know whether or not it is increased during the mucous metaplasia, hyperplasia, and/or hypertrophy commonly associated with inflammatory conditions in the airways [2]. To test the potential relationship in HBECCs between mucin stores and baseline secretion we compared the output of mucins from cultures grown under control conditions with those in which inflammatory mucous hyperplasia/hypertrophy was induced by a supernatant of mucopurulent material (SMM; [41–43]), collected and prepared from lungs removed from patients with Cystic Fibrosis during organ transplant surgeries. SMM is a protein-rich, incompletely characterized ‘broth’ containing a multiple cytokines, nucleotides, and proteases that together induce a massive upregulation of mucin gene expression and mucous hyperplasia in HBECCs (e.g., see Fig 5 in Ref [42]). A 72 h treatment of HBECCs with SMM caused a massive upregulation of secreted mucins (Fig 7). Visually, the surfaces of SMM-treated cultures appeared very ‘mucusy’, and the total mucins contained in the accumulated mucus removed from the cultures before the mucin secretion experiments was increased nearly 4-fold (Fig 7A). Consistent with the increase in mucin secretion implied by this result, both the mucins secreted under static conditions at baseline and in response to a maximal dose of ATPγS (100 μM) were also increased ~4-fold (Fig 7B). The increased levels of HBECC intracellular mucin stores [42] appears to be associated with increased rate of secretion, at both baseline and in response to agonist (Fig 7).

### Baseline secretion from mouse tracheas

The experiments with HBECCs above indicated that over macroscopic periods of time baseline mucin release is the predominant modality of secretion, that it is very sensitive to physical stresses, and that it may correlate with intracellular mucin stores. We used mouse models of mucin secretion to extend these findings, beginning with the Munc13-2 null mouse in which the accumulation of mucin in club cells under control conditions was hypothesized to be caused by a defect in baseline secretion [7]. If true, a decreased baseline secretion in the null mice might be expected; however, in our original studies, baseline mucin secretion rates appeared to be no different in the tracheas of WT and Munc13-2 null animals [7]. Therefore, a major goal of the present studies was to make a more definitive test of the hypothesis by minimizing potential experimental artifacts (next paragraph) and maximizing sensitivity of the mouse mucin detection assay (Methods, pages 4–5).

#### Perfusion effects in mouse tracheas

In the mouse tracheal perfusion protocol used in our laboratory [11], tracheas are equilibrated for 2 h at the same, low perfusion rates used during the experimental challenges (30 μl/min, ~51 mdyn/cm^2^). Mucin levels measured in the collected fractions begin at elevated levels and decline to a steady baseline rate of release over approximately the first 45 min of the 120 min equilibration period (see Fig 5D in Ref [11]). To test for effects of perfusion flow on measured baseline secretion, we adopted a stopped-flow procedure: mouse tracheas were equilibrated with constant perfusion for 2 h as usual, following which the perfusion was stopped for 30 min, then restarted and the perfusate collected into 5 min (~200 μl) fractions for an additional 35 min. Mucins released above the level of the perfused baseline during the period of reperfusion should reflect those secreted during the preceding period of stopped-flow.

**Fig 8:**
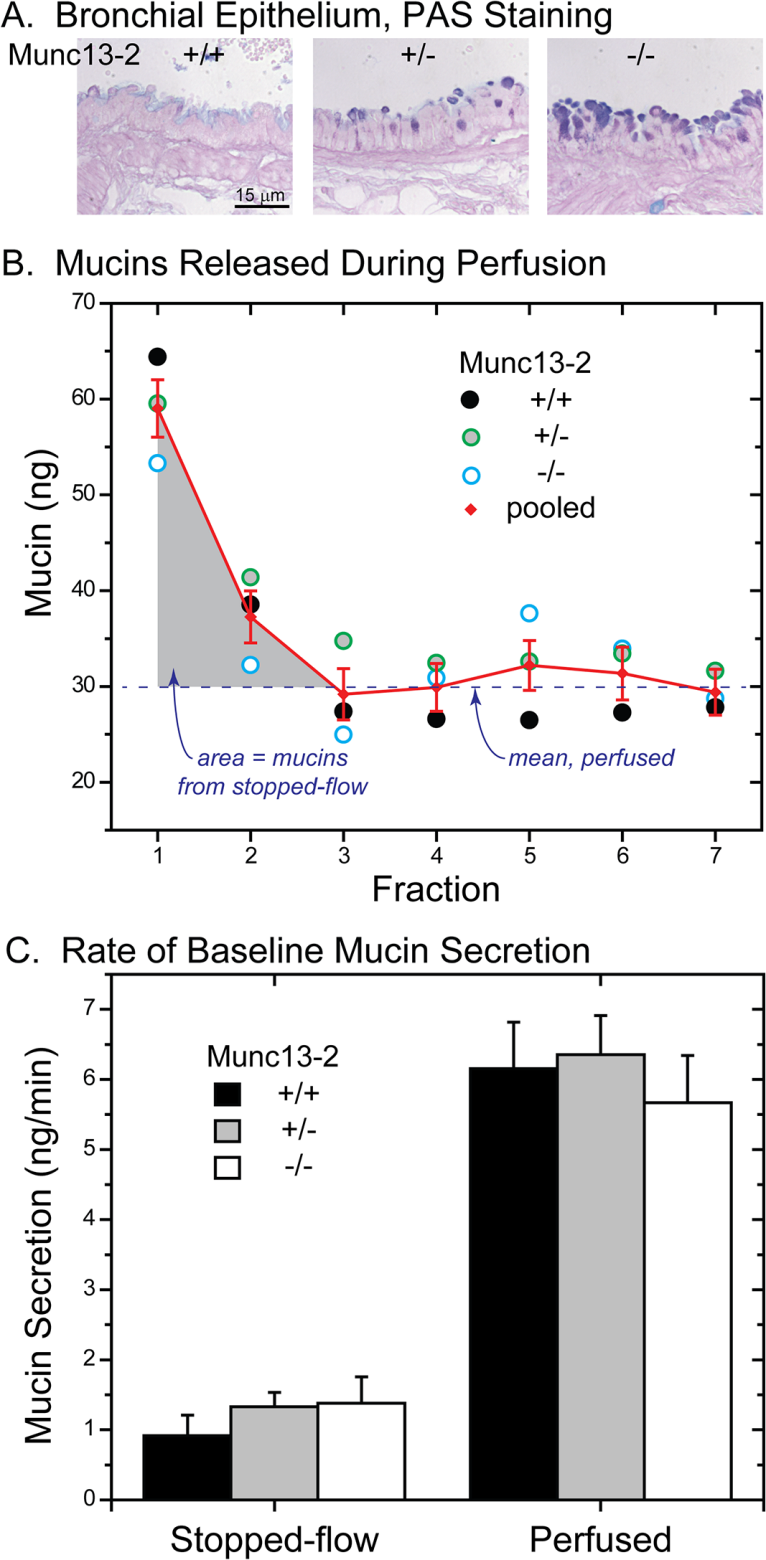
Baseline mucin secretion from Munc13-2 mouse tracheas. ***A*. *AB/PAS-stained sections of intrapulmonary bronchus***, illustrating the degree of intracellular mucin accumulation by genotype. ***B*. *Mucins released during tracheal perfusion*.** Following a 30 min period of stopped-flow, perfusion was restarted and 5 min (50 μl) fractions collected. Pooled data are indicated in red. Note the line labeled ‘mean, perfused’, indicating the mean level of mucins released during Fractions 3–7: the area indicated in light gray, above the line for Fractions 1 and 2, represents mucins released during stopped-flow. Mucins in all samples were determined by subunit ELISA. ***C*. *Baseline mucin secretion during stopped-flow and perfusion*.** Data from (*B)*, expressed as a rate. Despite the greater quantities of mucin released during stopped-flow *(B)*, the corresponding rate of release is lower due to the longer duration of the 30 min period (*vs*. 5 min fractions for the perfused period). Note the similarities in stopped-flow and perfused baseline secretion between genotypes (NS; n = 5), despite the progressive increases in mucin stores in the Munc13-2 deficient mice *(A)*.

Fig 8A shows a progressive increase in AB/PAS+ staining of the club cells in the tracheal epithelium of Munc13-2 mice, with essentially no staining in +/+ mice and intermediate and heavy staining in the Munc13-2 +/- and-/- mice, consistent with our original observations [7]. Fig 8B depicts the mucin secretion data for these mice, specifically, the mucins collected in each fraction from perfused tracheas of WT and Munc13-2 mice following the 30 min period of stopped-flow. Since data from the various Munc13-2 genotypes were similar and essentially overlaid one another, the data were pooled to better estimate the baseline-perfused mucin release (red data points and line, Fig 8B; n = 15). Note that the levels of released mucins were elevated in the first two fractions of reperfusion, and that for fractions 3–7 they lie on an obvious plateau (dotted line in the figure). The value of plateau, 30.01 ± 1.14 ng mucin per 5 min fraction, represents the average rate of baseline mucin release during perfusion for fractions 3–7. For fractions 1 and 2, the mucins released at levels above the plateau (Fig 8B, gray area), were taken to represent the mucins released during the preceding, 30 min period of stopped-flow. When the data from the stopped-flow and perfused components of baseline mucin secretion were so dissected and expressed as rates, baseline mucin release rates during perfusion exceeded the stopped-flow rates by ~4–6-fold (Fig 8C). Hence, restarting perfusion after a period of stopped-flow stimulates baseline mucin release. Notably, the ~4–6-fold increase in mouse tracheal mucin secretion caused by perfusion shear stresses is much larger than the effects of a maximal dose of purinergic agonist (ATPγS) applied during constant perfusion, which is generally ~2 – 3-higher than the perfused baseline [11].

Since the mucin release data from the different Munc13-2 genotypes were similar (Fig 8B), they could have been represented as single data set of stopped-flow and perfused baseline secretion in Fig 8C. We chose to treat them separately, however, to emphasize the fact that there were no differences in baseline mucin secretion in Munc13-2 deficient mice. These data are consistent with previous measurements which also failed to detect differences in perfused baseline mucin secretion levels in WT and Munc13-2 null mice [7]. Therefore, either our hypothesis concerning a defect in baseline secretion in Munc13-2 null mice [7] is incorrect, or the apparent lack of a difference indicates a compensating mechanism. A compensatory mechanism that would tend to equate the baseline rates of secretion in the different Munc13-2 genotypes would be a positive correlation of mucin secretion with the amount of mucin contained in intracellular stores, a relationship that would also explain the increases in HBECC mucin secretion during inflammation (Fig 7).

#### Mucin secretion vs. mucin stores: OVA-induced metaplasia

**Fig 9:**
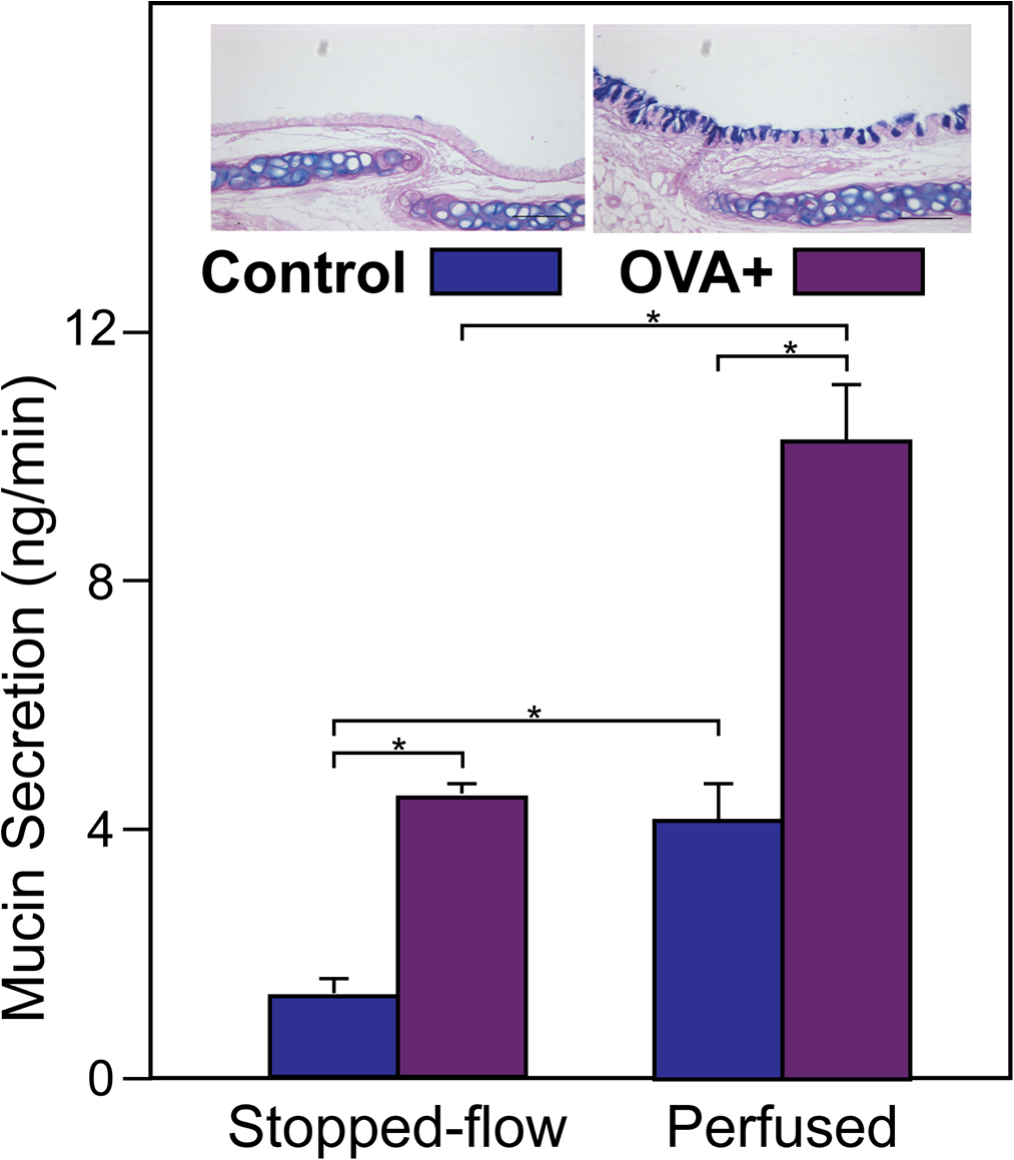
Effects of ovalbumin-induced mucous metaplasia on baseline mucin secretion from WT mouse tracheas. Tracheas from control and OVA-treated were equilibrated under continuous perfusion, and baseline mucin secretion was then determined under stopped-flow and following restoration of continuous perfusion, per Fig 2, using the subunit ELISA (* *p* < 0.05, n = 3). Insets at the top compare AB/PAS staining in sections of tracheas from control and OVA-treated mice.

As an initial test of a possible relationship between mucin stores and secretion, we measured baseline mucin secretion levels in WT mouse tracheas following induction of mucous metaplasia with ovalbumin. Relative to controls, OVA-induced mucous metaplasia increased mucin stores in club cells, as expected (Fig 9, top insets). Under stopped-flow conditions (Fig 9), OVA treatment caused a 4.0 ± 0.8 fold (n = 3) increase in baseline mucin secretion, relative to control, and under perfused conditions, baseline secretion was increased by 2.9 ± 0.7 fold (n = 3). Thus, the increased levels of mucin stores caused by major stimulation with an inflammatory insult do appear to correlate with higher baseline mucin secretion in mouse trachea, as they do in HBECCs (Figs 9 and 7).

#### IL-13-induced mucous metaplasia

**Fig 10:**
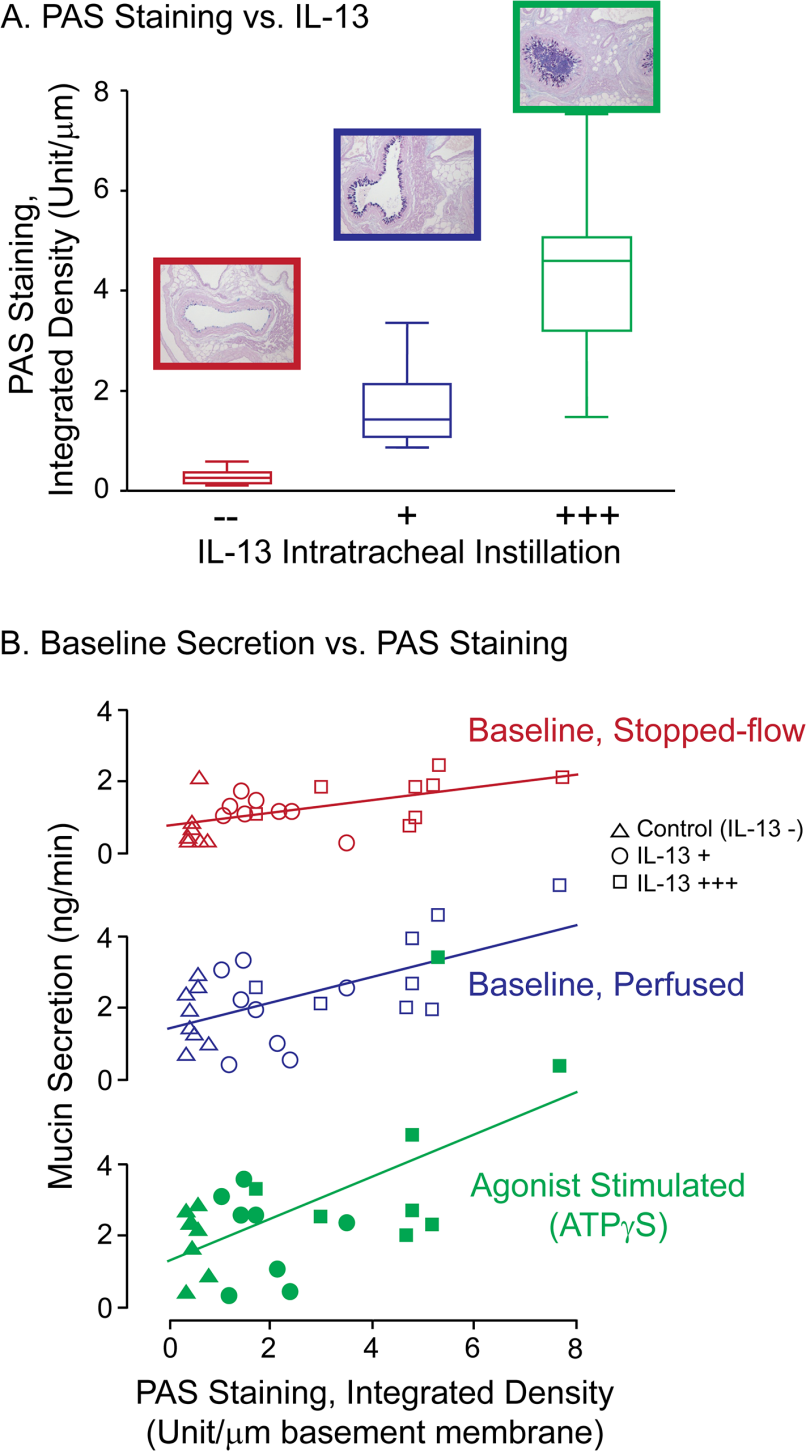
Effects of graded, IL-13 induced mucous metaplasia on WT mouse mucin stores and secretion. IL-13 (1 μg/instillation), or a sham, control solution, was instilled via the trachea into the airways of anesthetized mice, following Treatment protocols 0 (—), 1 (+), and 3 (+++) in Table 1. At the appropriate times following the last instillation, tracheas were harvested for mucin secretion experiments and the right lung lobe of each mouse was fixed for histology. ***A*. *IL-13 Effects on AB/PAS staining in the airways*.** Sections of the major intralobar bronchi were stained with AB/PAS and the degree of AB/PAS staining was quantified using ImageJ. Note the progressive mucous metaplasia and in Treatment 3 (+++) the presence of mucus plugs. The data are expressed as the integrated density per μm of basement membrane, using Box Plots (25^th^, 50 ^th^, 75 ^th^ percentiles). ***B*. *Relationship between AB/PAS staining and mucin secretion*.** Following IL-13 treatment, excised mouse tracheas were mounted for perfusion and equilibrated for 2 h, following which perfusion was stopped for the ‘Stopped-flow Baseline’, then restarted for the subsequent ‘Perfused Baseline’ and ‘Agonist Stimulated’ periods. The results are presented as scatter plots, with the measured mucin secretion rates, determined from subunit ELISAs, plotted against the AB/PAS Integrated Densities determined for the corresponding tissues from Panel A. Triangles, circles, and squares denote control and the 2 different IL-13 treatments, as indicated by the code, upper-right. Empty symbols denote secretions at baseline (stopped-flow and perfused) and filled symbols denote agonist-stimulated secretions. For the agonist-stimulated, IL-13+++ data (solid green squares), note the high value point that appears with the cluster of empty squares—to allow a simple visual comparison of the slopes, the scale for the agonist-stimulated data was not expanded to separate the data. All 3 slopes were significant, with correlation coefficients = 0.55, 0.61, and 0.59, and, with F ratio probabilities, *p <0*.*005*, *<0*.*002*, *and <0*.*001*.

To generate a mouse model with variable degrees of allergic mucous metaplasia, AB/PAS staining was examined in a set of mice treated by tracheal instillation with different amounts of IL-13, and/or for different times. Mice receiving 1 μg IL-13 on 1, 2, or 3 consecutive days, or 2 μg IL-13 for 2 consecutive days, were examined for the relative degree of mucous metaplasia on day 2, 3, or 5 (Table 1). Mucous metaplasia was determined by scoring both relative AB/PAS staining and degree of mucous plugging. Table 1 shows that increasing the amount of IL-13 instilled, the number of instillations, and the amount of time following the final instillation, all increased the degree of mucous metaplasia observed. Notably, severe mucous plugging was observed in Treatment #4, in which mice received 3 consecutive IL-13 instillations and were examined on day 5, instead of day 3 (S2 Fig). From these results, we chose to study mice for baseline secretion that received Treatments # 0 (control), 1, and 3. Using these protocols, Fig 10A shows that a positive correlation between the rigor of the IL-13 treatment and the degree of AB/PAS staining induced was achieved.

Tracheas harvested from the same mice studied in Fig 10A were perfused for baseline and agonist-stimulated mucin secretion. As in the earlier studies, the tracheas were equilibrated under continuous perfusion, perfusion was stopped for 30 min, restarted for 35 min, then the perfusate was switched to one containing ATPγS (100 μM) for a final 30 min period and the collected fractions were assessed for mucin content. Fig 10B shows the results as a series of scatter-plots depicting mucin secretion versus AB/PAS staining under the different perfusion regimes, with the control and increasingly rigorous IL-13 treatments indicated by different symbols. Note the strong correlation between the degree of mucous metaplasia and measured mucin release under each condition examined, i.e., baseline secretion, under both stopped-flow and continuous perfusion, and agonist-stimulated conditions. These data, in aggregate, therefore indicate that mucin release under baseline conditions is sensitive to the stresses imposed by perfusion flow, and that baseline and agonist-stimulated mucin secretion are both sensitive to the amount of mucin contained in intracellular mucin stores. Additionally, the data support the notion that the apparent lack of a mucin secretory phenotype in the Munc13-2 null mouse (Fig 8) may be due to the elevated levels of mucin stores in the tracheas of the Munc13-2 null mice that drive increased levels of mucin secretion, effectively masking the defect.

#### Perfusion effects in mice deficient for P2Y2R

**Fig 11:**
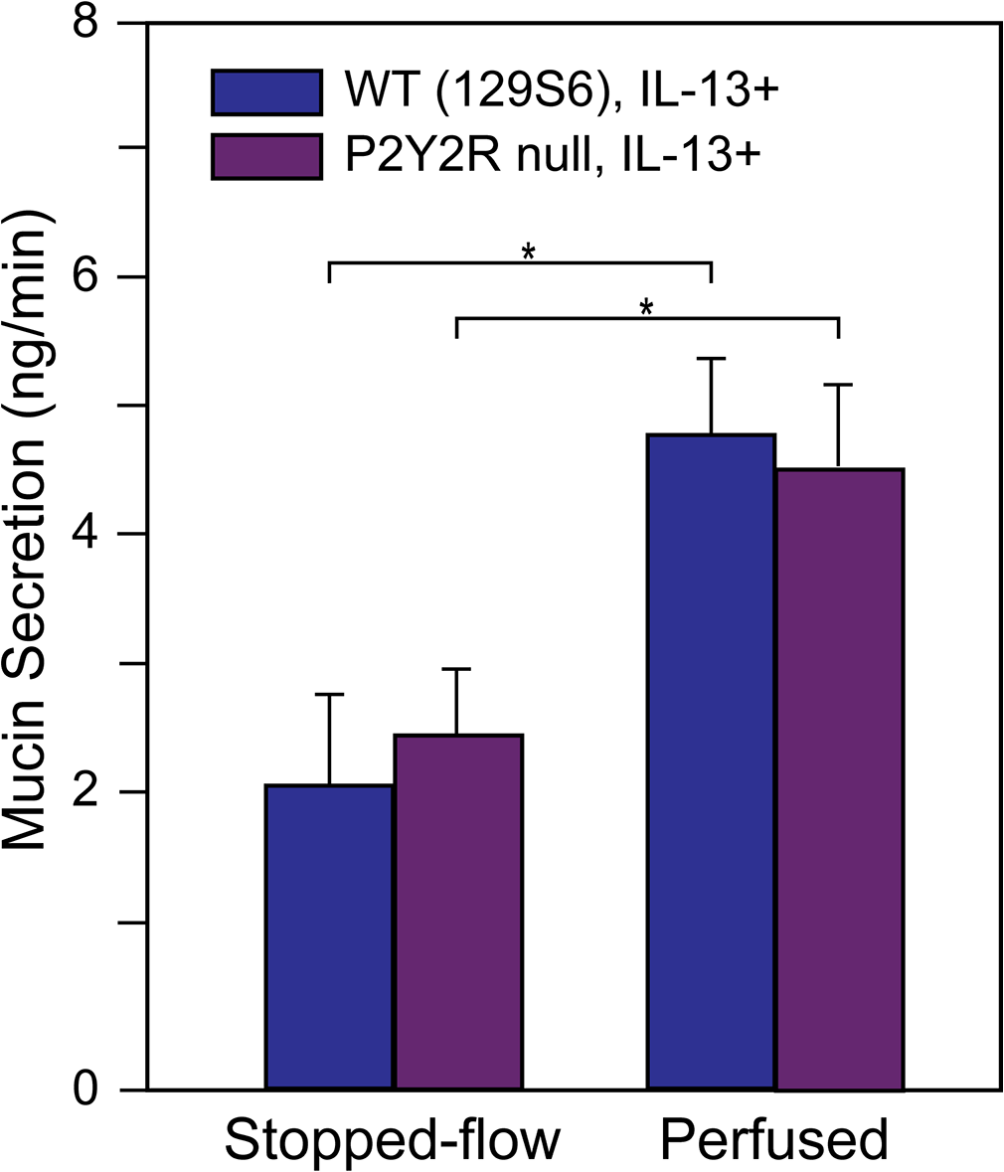
Persistence of perfusion effects in P2Y2R null mouse. After induction of mucous metaplasia with IL-13, tracheas from WT and P2Y2R null mice on the same 129S6 background were equilibrated under continuous perfusion, and baseline mucin secretion was then determined under stopped-flow and following restoration of continuous perfusion, from subunit ELISAs, per Fig 10 (* *p* < 0.05, n = 3 or 4).

Substantial evidence accumulated over the past two decades suggest that luminal purinergic signaling in the airways is responsible for maintenance of the airway surface liquid and mucus transport. Central to the signaling schemata proposed is the cellular release of ATP and its auto/paracrine feedback on the epithelium through P2Y_2_ purinoceptors (P2Y2R; [39,44,45]). To test whether the effects of stresses associated with perfusion of mouse tracheas on mucin secretion were mediated by this signaling system, we measured mucin secretion in tracheas from P2Y2R null mice, comparing them to WT 129S6 control mouse tracheas. As shown in Fig 11, there were no differences in baseline mucin secretion in the WT and P2Y2R null mice under stopped-flow and perfused conditions within the perfusion regime. Additionally, the increase in baseline mucin secretion when perfusion was re-started was also similar in the WT and P2Y2R null mouse tracheas, a result suggesting that perfusion effects on baseline mucin release are mediated by other, non-P2Y_2_ signaling events. For clarity, these results do not exclude the possible involvement of ATP or other purinergic agonists in the signaling events; they speak only against the involvement of the P2Y_2_ purinoceptor.

## Discussion

### Agonist-induced versus baseline mucin secretion

A well-stained goblet cell in a histologic slide is impressive for the quantity of its stored mucins, with the stores generally occupying the major fraction of the total cell volume (e.g., Figs 2, 9 and 10). Such observations sometimes lead to suggestions of goblet cells acting as sentinels, holding mucin stores for acute release at a critical time in response to humoral or other cues. This notion is reinforced by observations of living airway goblet cells in epithelial explants from canine trachea and human nasal epithelia by differential interference contrast microscopy. For goblet cells at rest, the rates of mucin granule exocytosis are very low: in 75 cells observed for an aggregate period of 24.3 h, the average rate of exocytic events per cell was 0.05 EE/min [33,40]. These cells then responded to a maximal concentration of ATP (100 μM) with a short,<1 min, burst of exocytoses with maximal rates of 87.9 (canine) and 118.2 (human) EE/min, respectively, followed by a long-lasting plateau with much lower rates (1.9 EE/min in canine trachea). In both studies, the goblet cells were totally, or nearly depleted in the first few minutes of exposure to agonist [33,40], consistent with the observations in HBECCs that mucin release is complete in <5 min (Fig 6), and that following release the goblet cells are fully depleted of their mucin stores (Fig 2; [15]). In fact, the acute emptying of mucin stores is likely of pathophysiologic importance in causing acute airway obstruction in patients suffering inflammatory airways diseases, and in concept it could lead to the formation of excessively concentrated mucus by overwhelming the ambient surface liquid. Such pathologies may occur, for example, in patient airways with chronic bronchitis, status asthmaticus, and cystic fibrosis [2,46].

Although the release of mucins under baseline conditions is not nearly as dramatic as the acute exocytic response of goblet cells to agonist, baseline secretion may be the more important mode of mucin release over the long term. With the simplifying assumptions that baseline secretion is constant and that the mucins secreted by agonist exposure are limited to one bolus release per day by a limited goblet cell synthetic capacity (Fig 2), baseline mucins exceeds those released acutely by several fold (Fig 1B). An interesting feature of baseline mucin secretion is that it appears to be sensitive to, and is possibly dependent upon, mechanical stresses (Figs 3–5 and 8–11). Further, with HBECCs the mechanical stresses of handling yielded what appeared to be unitary baseline mucin secretory responses (Figs 3 and 4), possibly indicating a fundamental control mechanism. Thus, our view of the airway goblet cell phenotype may require a substantial readjustment, for the data suggest that one of their primary functions is the production and release of mucins at baseline.

In principal, a goblet cell, or a club cell at steady-state can secrete mucins at the same rate as they are synthesized, fully independent of mucins sequestered in a storage pool. Hence, their baseline secretory activity may be critical and their mucin stores, when present, considered a reserve held for acute release. These notions are supported by our recent observations in adult mouse airways, [i] that club cells secrete Muc5b at baseline even though mucin stores under control conditions are not apparent by AB/APS staining [7,47], and [ii] that continuous mucus production is required for airway defense in the mouse [48].

### Potential pathways for baseline mucin secretion

In airway goblet cells, the purinergically stimulated, Ca^2+^-dependent exocytosis of mucin granules occurs via the classical, regulated secretory pathway [5,49,50]. In other secretory cells, however, there is also a less well-known branch of the regulated secretory pathway, the ‘basal’ or ‘constitutive-like’ secretory pathway. It is suggested by studies demonstrating that cargo typically processed via the regulated secretory pathway is released exocytically from non-stimulated cells [5,51–53]. Interestingly, ≥90% of von Willebrand factor, a similarly large glycoprotein with strong homologies to polymeric mucins [54], is secreted from the vascular endothelium, not by the exocytic release of Weibel-Palade bodies, but by the basal secretory pathway [17]. Hence, there is a good chance that a basal secretory pathway in goblet cells mediates baseline mucin release either in part, or its entirety.

The final steps of regulated exocytosis consists of a sequence of highly specific protein-protein interactions beginning with Rab3/27 and Munc13 mediated tethering of a vesicle or granule to Munc18 or other SM protein defining an exocytic docking site, followed by assembly of the SNARE complex, and ending with synaptotagmin/Ca^2+^-stimulated exocytic fusion and cargo release [55,56]. Each of the regulatory and SNARE proteins in the chain represents a family with multiple isoforms. Data from genetically modified mice have generally indicated the participation of at least two isoforms of each exocytic protein family in secretion. For instance, knockout of Munc13-2 causes mucins to accumulate in club cells in the mouse airways, suggesting a defect in baseline secretion, but agonist-stimulated secretion is unaffected [7]. Similarly, knockout of the Rab27 effector protein Slp2-a [57], or of Syt2 [58], affects either baseline or agonist-stimulated mucin secretion, but not both. Knockout of VAMP8 strongly inhibits agonist-stimulated mucin secretion [59] and mucins accumulate at baseline; however the baseline accumulation phenotype is distinctly less than that observed in Munc13-2 null mice on the same genetic background (Y Zhu and CW Davis, unpublished observations). Importantly, none of the airway mucin secretory phenotypes in the mouse knockout models observed to date resulted in a complete failure of mucin secretion. The results therefore suggest complicated scenarios, e.g., independent SNARE pathways for baseline and agonist-stimulated mucin secretion, a single SNARE pathway regulated differentially for baseline and agonist-induced secretion, or a pathway(s) endowed with high degree of redundancy.

### Shear stress-dependence of baseline secretion

Mucin secretion at baseline *in vivo* occurs in a mechanically dynamic lung environment: goblet cells are exposed continuously to mechanical forces that may stimulate and/or influence the production and secretion of mucins. These forces include shear stresses from air drag during tidal breathing and cough, compressive stresses due to chest cavity pumping, and there may be stresses arising from neighboring ciliated cells which are obviously very dynamic. Such mechanical forces have many, diverse effects on the lung at the cellular level [60], and recently they have been shown to have profound effects on the regulation of the airway surface liquid by stimulating the release of ATP into the lumen [38,61,62]. In this paper we show that baseline mucin secretion from both native mouse trachea and HBECCs is not only highly sensitive to the shear stresses associated with luminal perfusion, but may also be dependent on shear stresses and/or other mechanical stimuli. In HBECCs subjected first to a Careful Wash procedure to remove accumulated mucus, regular, periodic sampling lead to an seemingly stable baseline rate of mucin secretion; yet, when the middle sampling period was lengthened from 1 to 72 h, the apparent rate of mucin secretion was significantly depressed (Fig 3B), suggesting that the secretion observed was induced by the mechanical stimulation associated with the sampling procedure. This possibility was confirmed by the subsequent finding that mucin release relaxes exponentially following a luminal wash, with a half-time of approximately 2.75 h (Fig 4). Significantly, mucin secretion declined to near zero rates in the experiments of Fig 4, suggesting that mechanical stresses may be essential in the lung for stimulating baseline mucin production and secretion.

The stopped-flow perfusion experiments with mouse trachea generally supported the notion of shear stress-induced baseline mucin release: the rates of release following restoration of perfusion were ~6-fold above those measured during stopped-flow (Fig 8C). Mucin release during stopped-flow was determined as the mucins secreted above the perfused-baseline rate, but in light of the immediacy of the mucin secretory response to jumps in flow rate for HBECCs (Fig 5A) there is a possibility that some of the supra-baseline release of mouse mucins immediately following restoration of tracheal perfusion was in fact due to a similar response to shear-stress. In other studies with HBECCs and airway cell lines, the onset of shear causes a spiking release of ATP that is Ca^2+^-dependent, with a Ca_i_^2+^ signal that has similar time course [36,38,63]. In fact, the time courses of both ATP release and intracellular Ca^2+^, are very much like the mucin secretory response we observed with HBECCs (Fig 5A). Given the Ca^2+^-dependence of mucin secretion [49], it is therefore likely that the mucin secretory response of HBE goblet cells to shear stress is triggered by Ca^2+^, and it is possible, though not necessary, that it is mediated by ATP. Although HBE cells secrete ATP in response to shear stress, in fact, the concentrations of ATP measured within the periciliary environment of HBECCs following the phasic shear stresses <100 mdyn/cm^2^ were ~30 nM, or less [38], well below levels known to stimulate IP_3_ production, Ca^2+^ mobilization, or mucin secretion [9,64]. The shear stress-induced mucin secretory response of P2Y2R null mice was intact (Fig 11), which supports the notion of a signaling system independent of P2Y2R. This result is not conclusive, however, as mice with mucous metaplasia are responsive to adenosine, via the A3 receptor [65]. Were the shear stress to cause the release of ATP, its resulting ecto-metabolism to adenosine could have been stimulatory. In any case, the sensor of shear stresses for goblet cells is unknown and the possibilities include goblet cell mechanosensitive channels or receptors, autocrine factors, and paracrine factors from ciliated cells.

### Store-dependence of mucin secretion

One of the more interesting observations of these studies was the apparent positive relationship between mucin store volume and levels of baseline and agonist-induced mucin secretion. In mice with ovalbumin-induced mucous metaplasia, both stopped-flow and perfused baseline mucin secretion rates were elevated (Fig 9), and a graded-dosing with IL-13 caused proportional increases in club cell mucin stores and the rates of baseline and agonist-induced mucin secretion (Fig 10). Similarly, in HBECCs treated with SMM, total mucin production and the rates of baseline and agonist-induced mucin secretion were all increased (Fig 7). It is tempting to think of this positive relationship in thermodynamic terms, i.e., as though the rate of mucin secretion were higher because of a stronger driving force from the increase in mucin stores. Mucin secretion, however, is not a dissipative process. Rather, the secretory pathway is highly energy dependent, with each step of the process, including mucin transcription, translation, vesicle transport, post-translational processing, packaging, and secretion, all being dependent on ATP hydrolysis. Additionally, each step is tightly regulated. Hence, the positive relationship that appears to exist between the quantity of mucin stores and rate of mucin secretion implies the existence of a tight, negative feedback relationship between mucin stores and secretion, complete with suitable sensors and signaling pathways—all of which remain to be identified.

In all of the common inflammatory airways diseases inflammation drives an increase in mucin production and secretion. Much effort has gone into understanding the role of mucin gene transcription in this process [46,66,67], but too little attention has been paid to the regulation of mucin biosynthesis downstream of transcription or to the attendant changes that are likely to occur in the secretory pathway, such as an expansion of the ER and Golgi compartments. These aspects of mucin synthesis are important in their own right, but with realization of the importance of baseline secretion and its dependence on mucin store volume, understanding the interrelationships and their regulation takes on an added urgency. These questions, as well as many others pertaining to the quantitative and regulatory relationships along the mucin biosynthetic pathway and its secretion require a directed effort to elevate our knowledge, to drive the development of pharmaceutic interventions that might benefit patients suffering from obstructive airways diseases.

## Supporting Information

S1 File Abbreviations and definitions. (DOCX)

S1 Fig Perfusion plug and gasket with perfusion slot for Transwell. HBECCs grown in TClears were perfused using a Neoprene gasket (2 mm thick) with an oval cutout to from a perfusion slot. The gasket was pressed down onto the luminal surface of the HBECC and held in place with a ‘perfusion plug’, which possessed inflow and outflow channels and an O-ring seal, as depicted.
(PDF)

S2 Fig Severe mucous plugging in WT mice treated rigorously with IL-13. Mice received Treatment #4 from Table 1: IL-13 (1 μg) was instilled into tracheas of isoflurane-anesethesized mice on Days 0, 1, and 2, and the mice were euthanized for tissue harvest on Day 5. These images typify bronchial airways that are severely plugged, some, as in *A*, for considerable lengths. Others, however, have open lumens, as in *C*. NOTE: Before euthanasia, the mice with this most rigorous IL-13 treatment used, were apparently normal in all respects, despite the high degree of mucous plugging. Possible reasons for the apparent good health include the lack of infection and the fact that the mucus plugs were newly formed.
(PDF)
