# Supplementary material for: Baseline Goblet Cell Mucin Secretion in the Airways Exceeds Stimulated Secretion over Extended Time Periods, and Is Sensitive to Shear Stress and Intracellular Mucin Stores
Source: PLoS One. 2015 May 29;10(5):e0127267. doi: 10.1371/journal.pone.0127267 (PMC4449158; doi:10.1371/journal.pone.0127267)
Supplement: S1 File — (DOCX) [file pone.0127267.s001.docx]

**Abbreviations and Definitions**

AB/PAS, alcian blue, periodic acid-Schiff stain

AB/PAS+, cells stained positively by AB/PAS

ALI, air-liquid interface

BEGM, bronchial epithelial cell growth medium

‘Careful Wash’, the series of 4, rigorously controlled luminal washes that removed mucus from HBECCS prior to a mucin secretion experiment

EE, exocytic event

HBE cells, human bronchial epithelial cells

HBECC, human bronchial epithelial cell culture

OVA, ovalbumin

WT, wildtype

TGN, trans Golgi network;
