# Supplementary material for: Baseline Goblet Cell Mucin Secretion in the Airways Exceeds Stimulated Secretion over Extended Time Periods, and Is Sensitive to Shear Stress and Intracellular Mucin Stores
Source: PLoS One. 2015 May 29;10(5):e0127267. doi: 10.1371/journal.pone.0127267 (PMC4449158; doi:10.1371/journal.pone.0127267)
Supplement: S1 Fig — HBECCs grown in TClears were perfused using a Neoprene gasket (2 mm thick) with an oval cutout to from a perfusion slot. The gasket was pressed down onto the luminal surface of the HBECC and held in place with a ‘perfusion plug’, which possessed inflow and outflow channels and an O-ring seal, as depicted. (PDF) [file pone.0127267.s002.pdf]

**Figure S1. Perfusion plug and gasket with perfusion slot for Transwell.**

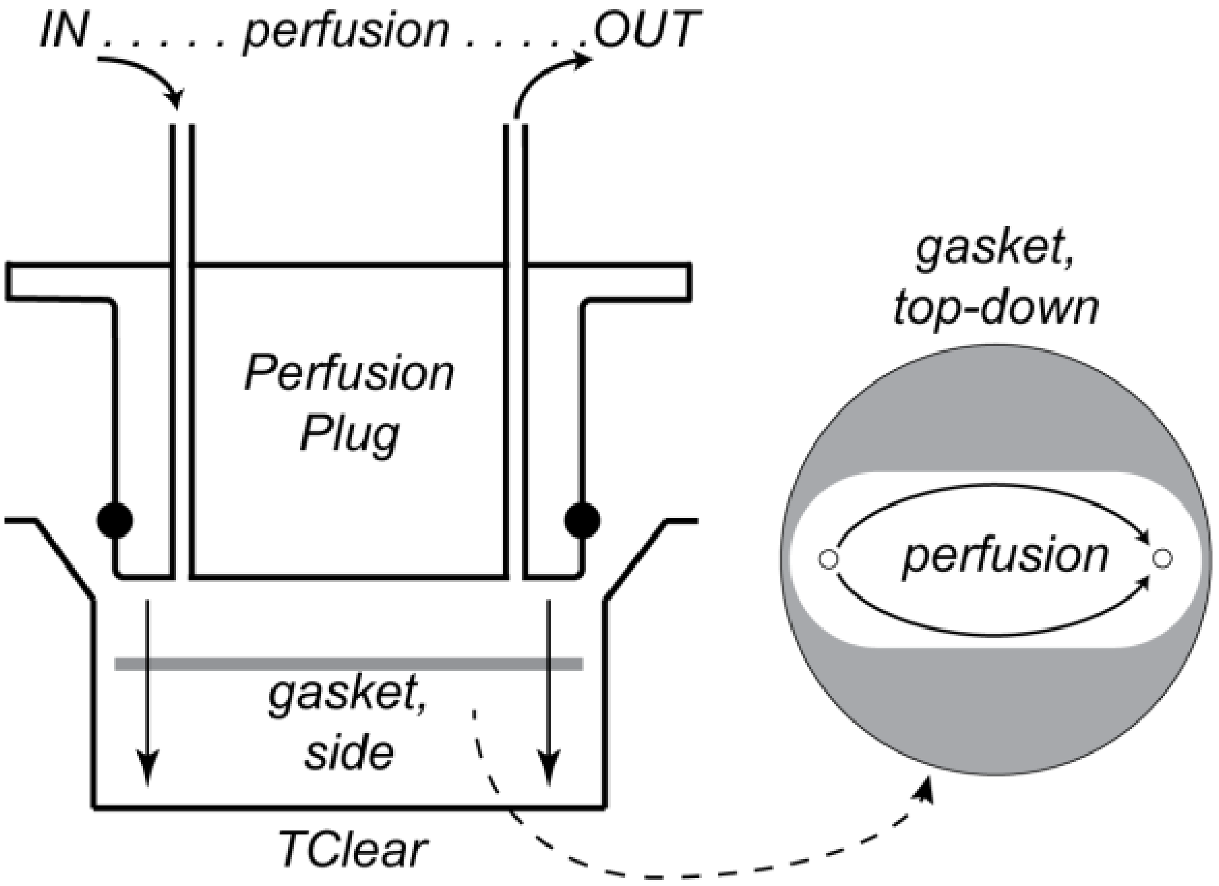


**Fig S1. Perfusion plug and gasket with perfusion slot for Transwell** HBECCs grown in TClears were perfused using a Neoprene gasket (2 mm thick) with an oval cutout to from a perfusion slot. The gasket was pressed down onto the luminal surface of the HBECC and held in place with a 'perfusion plug', which possessed inflow and outflow channels and an O-ring seal, as depicted.
