# Supplementary material for: Baseline Goblet Cell Mucin Secretion in the Airways Exceeds Stimulated Secretion over Extended Time Periods, and Is Sensitive to Shear Stress and Intracellular Mucin Stores
Source: PLoS One. 2015 May 29;10(5):e0127267. doi: 10.1371/journal.pone.0127267 (PMC4449158; doi:10.1371/journal.pone.0127267)
Supplement: S2 Fig — Mice received Treatment #4 from Table 1: IL-13 (1 μg) was instilled into tracheas of isoflurane-anesethesized mice on Days 0, 1, and 2, and the mice were euthanized for tissue harvest on Day 5. These images typify bronchial airways that are severely plugged, some, as in A, for considerable lengths. Others, however, have open lumens, as in C. NOTE: Before euthanasia, the mice with this most rigorous IL-13 treatment used, were apparently normal in all respects, despite the high degree of mucous plugging. Possible reasons for the apparent good health include the lack of infection and the fact that the mucus plugs were newly formed. (PDF) [file pone.0127267.s003.pdf]

Figure S2. Severe mucous plugging in WT mice treated rigorously with IL-13.

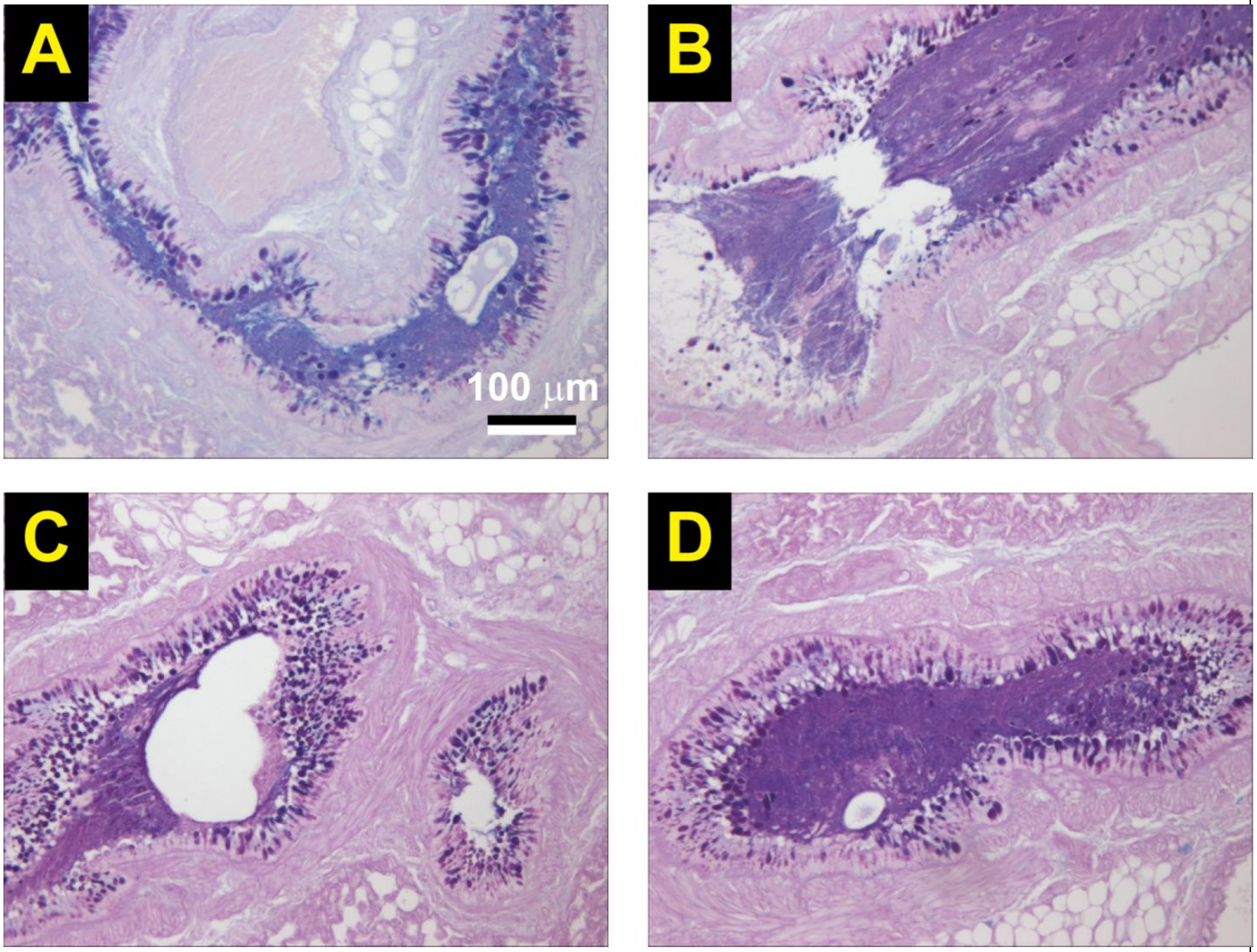

Fig. S2. Severe mucous plugging in WT mice treated rigorously with IL-13. Mice received Treatment #4 from Table 1: IL-13 (1 μg) was instilled into tracheas of isoflurane-anesethesized mice on Days 0, 1, and 2, and the mice were euthanized for tissue harvest on Day 5. These images typify bronchial airways that are severely plugged, some, as in *A*, for considerable lengths. Others, however, have open lumens, as in *C*.

NOTE: Before euthanasia, the mice with this most rigorous IL-13 treatment used, were apparently normal in all respects, despite the high degree of mucous plugging. Possible reasons for the apparent good health include the lack of infection and the fact that the mucus plugs were newly formed.
